# Internal defect scanning of sweetpotatoes using interactance spectroscopy

**DOI:** 10.1371/journal.pone.0246872

**Published:** 2021-02-09

**Authors:** Michael W. Kudenov, Clifton G. Scarboro, Ali Altaqui, Mike Boyette, G. Craig Yencho, Cranos M. Williams

**Affiliations:** 1 Department of Electrical and Computer Engineering, North Carolina State University, Raleigh, North Carolina, United States of America; 2 Department of Biological and Agricultural Engineering, North Carolina State University, Raleigh, North Carolina, United States of America; 3 Department of Horticultural Science, North Carolina State University, Raleigh, North Carolina, United States of America; Politecnico di Milano, ITALY

## Abstract

While standard visible-light imaging offers a fast and inexpensive means of quality analysis of horticultural products, it is generally limited to measuring superficial (surface) defects. Using light at longer (near-infrared) or shorter (X-ray) wavelengths enables the detection of superficial tissue bruising and density defects, respectively; however, it does not enable the optical absorption and scattering properties of sub-dermal tissue to be quantified. This paper applies visible and near-infrared interactance spectroscopy to detect internal necrosis in sweetpotatoes and develops a *Zemax* scattering simulation that models the measured optical signatures for both healthy and necrotic tissue. This study demonstrates that interactance spectroscopy can detect the unique near-infrared optical signatures of necrotic tissues in sweetpotatoes down to a depth of approximately 5±0.5 mm. We anticipate that light scattering measurement methods will represent a significant improvement over the current destructive analysis methods used to assay for internal defects in sweetpotatoes.

## 1.0 Introduction

Measuring the internal characteristics of samples, non-destructively, is of critical importance in biomedical diagnostics [1–3] and is an emerging optical detection method for food sorting and quality assessment [4]. Current methods for grading horticultural products focus on culling based on incorrect size and shape [5–7] in addition to higher-order effects, such as external defects or defects close to the skin’s surface. Higher-order effects and blemishes include bruising [8], disease or insect damage [9, 10], residues related to food safety [11], firmness [12], and soluble solids [13]. Many of these techniques use hyperspectral or multispectral cameras, built using various sensor architectures [14]. Meanwhile, deeper internal defects can be sorted in some commodities, including hollow heart in potato, which is identified using X-ray scanners [15]. While X-ray scanning may appear to be the most logical choice for detecting all internal defects, it is limited to quantifying only density changes. Other changes, such as color or spectral changes caused by tissue necrosis, do not create a change in density. Thus, an intermediate technique is generally needed to obtain visible and near-infrared measurements of tissues beyond what typical reflectance spectroscopy offers.

Light scattering spectroscopy, of which interactance spectroscopy can be considered a subset [16–18], has been deployed in biomedical imaging for disease diagnosis based on cellular size distributions [1, 19]. These scattering-based measurements are emerging as a technique to quantify deeper tissues in horticultural crops [4, 20, 21]. In light scattering spectroscopy, light is “injected” into the sample to quantify its optical scattering coefficients [3]. The illumination light can be continuous wave [22, 23], temporally modulated or pulsed [24, 25], or spatially modulated [26, 27], and can be categorized into spatially-resolved, time-resolved, or frequency-domain techniques [4]. The scattering and absorption properties of the underlying tissues are then quantified, which can then be used directly for diagnosis, or they can be further processed to provide estimates of, *e*.*g*., scattering particle (cellular) size distributions [28–31]. Scattering coefficients have also been quantified in potato, which we leverage to some extent in the current study [32]. A similar strategy to light scattering spectroscopy is interactance spectroscopy, which collects light that has been deeply scattered within the tissues. Such configurations often deploy a second slit to reject the light that is reflected off the target’s surface [16]. In interactance spectroscopy, the results are less quantitative concerning scattering particle size distributions and absolute absorption; instead, measurements represent relative spectral absorption within internal tissues [33].

In this paper, we investigated the feasibility of continuous-wave interactance spectroscopy, using a fixed distance probe, to quantify internal necrosis (IN) non-destructively. Our primary goal in making these measurements was to quantify relative spectral absorption characteristics, during on-line sorting, to improve product quality [34]. In section 2, we overview the experimental methods of interactance spectroscopy implemented in the current study, including a schematic of the experimental setup. In section 3, we overview experimental interactance spectra of healthy and necrotic tissues, transmission measurements of the skin tissue, and interactance spectra of necrotic tissue found in four cut sweetpotatoes. In section 4, we detail an optical scattering model that was deployed in the *Zemax* optical design and ray tracing program and compare these theoretical models to experimental results. Finally, in section 5, we discuss the results, limitations, and future work and conclude in section 6 to summarize our results. Through this work, we aim to develop a new method of deploying interactance spectroscopy in high-throughput sweetpotato sorting and packing facilities.

## 2.0 Materials and methods

### 2.1 Light scattering measurement setup

**Fig 1:**
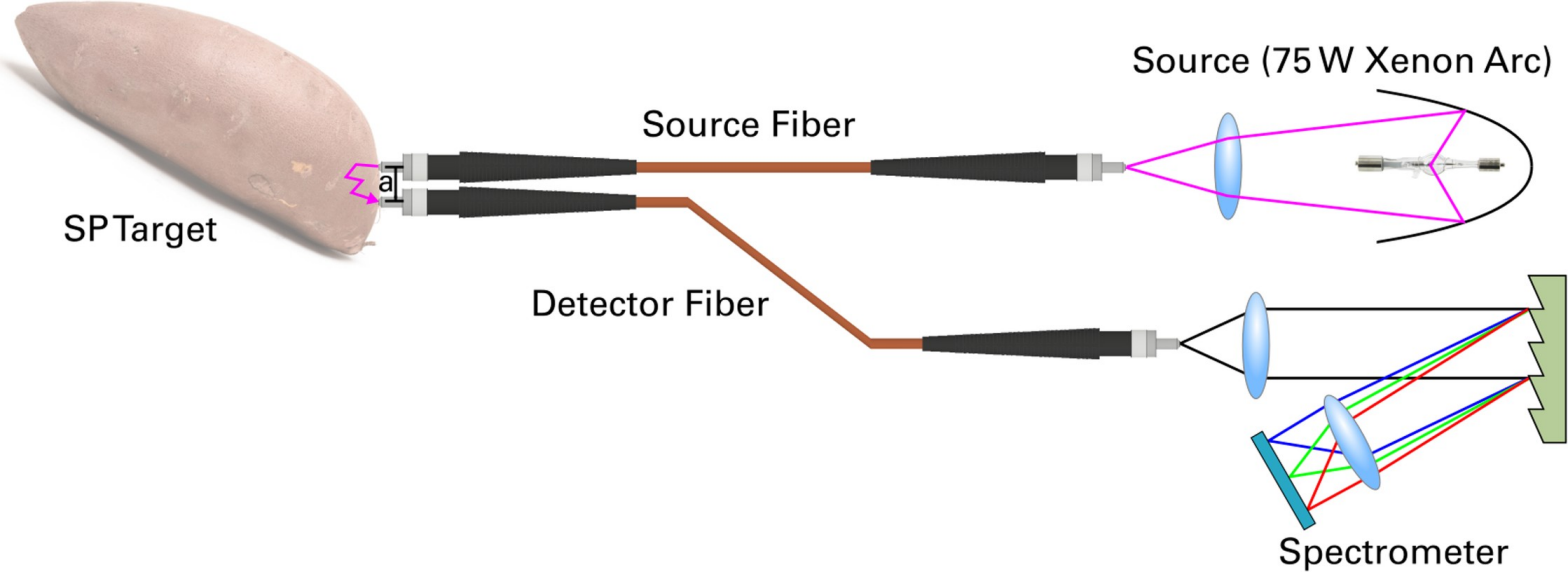
Schematic overview of the interactance spectroscopy setup. Optical fibers were placed directly onto the sweetpotato target that was positioned a distance *a* apart.

Interactance spectra were measured using the experimental configuration depicted in **Fig 1**. Light from a 75 W xenon arc lamp was coupled into a source fiber positioned a distance *a* away from a detector fiber. Here, *a* is the center-to-center distance between the fiber’s cores and was 8.5 mm for all measurements. The core diameter of each fiber was 1 mm. Both fibers were placed into direct contact with the sweetpotato (SP) tissue, such that the detector fiber can collect multiply-scattered photons. This light was then coupled into a spectrometer (Ocean Optics USB2000, 600–1000 nm), which recorded the scattered light on a computer. Interactance spectra were then post-processed using *Matlab 2018b* following the subsequent procedures’ equations.

In *Matlab*, the USB2000 spectrometer was spectrally calibrated using emission lines from helium and argon gas-discharge lamps. Spectra were then interpolated onto this calibrated wavelength axis using linear interpolation. Finally, it should be noted that all measured data of SPs with IN were collected from the orange-fleshed and red-skinned *Covington* variety, which comprises approximately 90% of North Carolina’s current acreage.

### 2.2 Pure healthy and necrotic tissues

**Fig 2:**
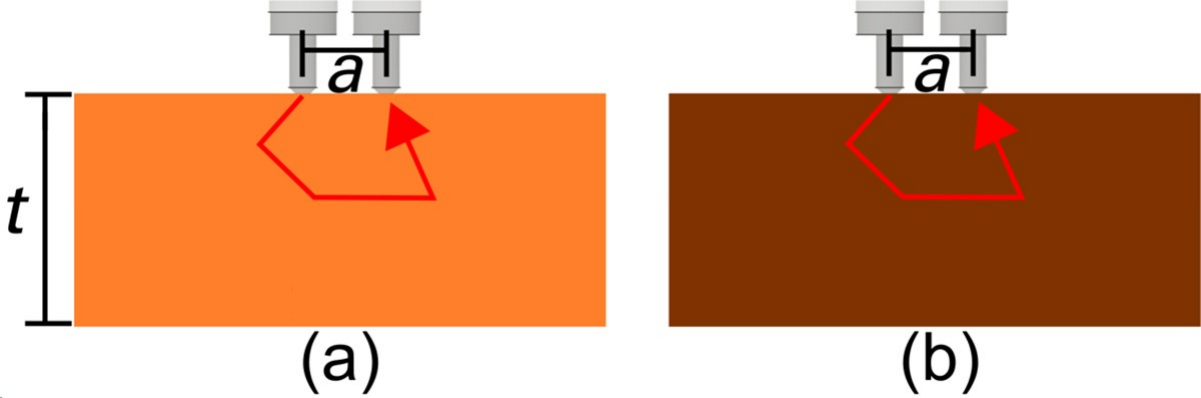
Experimental setup. A cut tissue section from one sweetpotato was measured with the optical fibers, which were placed into direct contact with the (a) healthy or (b) necrotic tissues.

Tissues from one SP with IN were taken destructively. The SP was sliced into several 10 mm thick sections, and pure healthy and necrotic tissue was measured as detailed below in **Fig 2**(A) and **2**(B), respectively, in which the probe was placed into direct contact. In this geometry, scattered light interacts only with the healthy or necrotic tissue slab of a given thickness *t*. For necrotic tissue, *t* was limited to a maximum of approximately 5 mm due to the samples we had available, while for healthy tissue, we used a thickness of 10 mm.

For each interactance spectrum that was collected, we implemented the following procedure: (1) A dark spectrum (*I*_*Dark*_) was captured in which the fiber probe was turned on but pointed at a distant (> 3 m) black (reflectivity < 2%) surface; (2) A scattering spectrum (*I*_*Scattering*_) was measured by placing the probe against the SP’s tissue and integrating the scattered light for 2 seconds; and (3) A incident light spectrum (*I*_*Lamp*_) was taken by measuring a white (99% reflectivity) spectralon tile, placed 200 mm away from the fiber probe, to measure the xenon arc lamp’s input spectrum. Interactance spectra were calculated by

$$I_{IS}=\left(I_{Scattering}-I_{Dark}\right)/\left(I_{Lamp}-I_{Dark}\right),$$ (1)

where all intensities are implicitly dependent on wavelength *λ* for clarity. Finally, all measured or simulated interactance spectra are normalized to 825 nm to aid in interpretation, such that

$$I_{IS,norm}=I_{IS}/I_{IS}\left(825\;\mathrm{nm}\right).$$ (2)

### 2.3 Internal necrosis measurements through the skin

**Fig 3:**
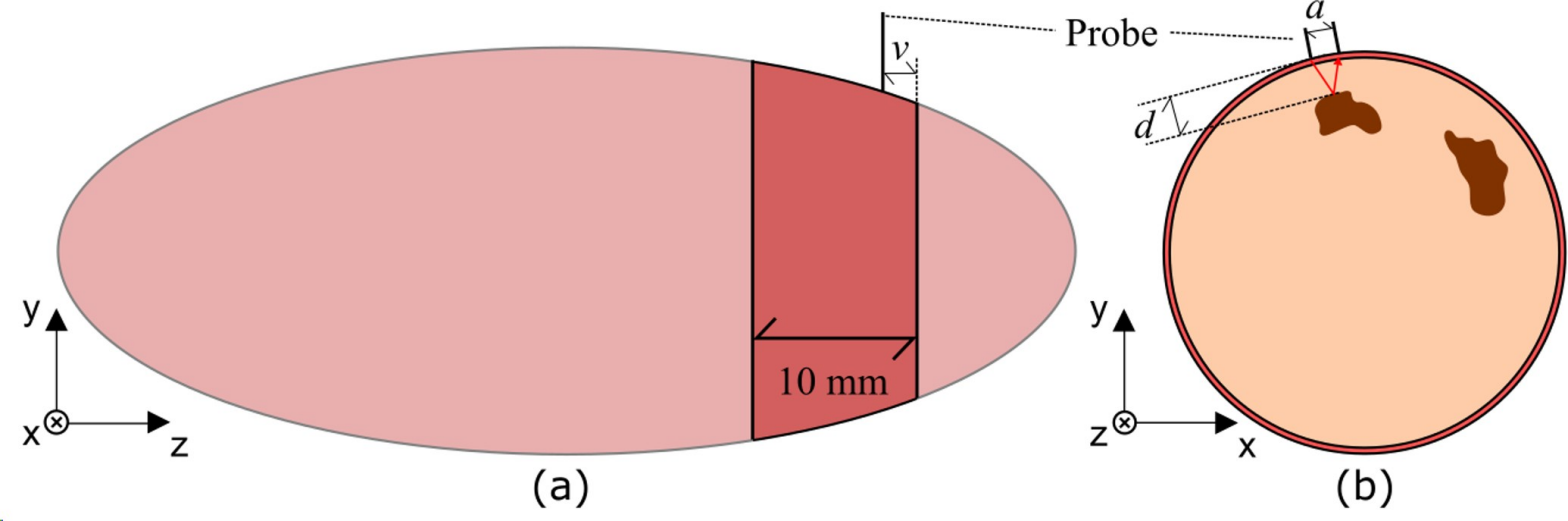
Interactance spectrum sampling of tissue slices. (a) Sweetpotatoes were sampled towards their proximal ends in approximately 10 mm thick sections. (b) The probe was positioned adjacent to the necrotic tissue, and the depth to the defect was measured.

Due to a limited number of SPs with IN that we could identify among stakeholders, a total of 16 spectra were collected from 6 different SPs containing skin in which one spectrum was collected at each measurement position. Cross-sections, with a thickness of approximately 10 mm, were taken towards the proximal end, as depicted in **Fig 3(A)**. These slices were then sampled with the interactance probe in regions containing IN, as depicted in **Fig 3(B),** where the defect’s depth *d* was measured using calipers. Slices were then measured with the probe placed close to the slice’s edge, such that the distance *v* in **Fig 3(A)** was approximately 1 mm. Since IN changes depth slowly as a function of *z* position [34], this ensured that *d* remained representative of the necrotic tissue’s depth. Interactance spectra were collected according to the 3 step procedure detailed previously in section 2.2 for pure healthy and necrotic tissues, except that in step 2, the light was integrated for 10 seconds. Interactance spectra were subsequently calculated using Eq (1).

### 2.4 Measuring the spectral variability among sweetpotatoes

A total of 22 randomly selected whole *Covington* SPs were measured. First, the SPs were divided into two batches containing a total of 10 and 12 samples each. Each batch was then laid out on a black posterboard for conventional three-color (red, green, and blue–or RGB) photography. A total of two images were collected from each batch to capture two sides of each sample. Using the SP’s orientation in this image as a reference, each SP was then divided lengthwise into a proximal, middle, and distal region, each comprising 1/3^rd^ of the total SP’s length. Within each region, a total of 4 interactance spectra were collected from four randomly selected spatial points. This procedure produced 12 total interactance spectra for each SP sample.

For each whole SP, interactance spectra were collected using the following procedure: (1) A dark spectrum was captured in which the fiber probe was turned on but pointed at a distant (> 3 m) black (reflectivity < 2%) surface; (2) The sequences of 4 interactance spectra were collected, within each of the 3 spatial regions, using an integration time of 250 ms; and (3) A spectrum of a white (99% reflectivity) spectralon tile, placed 200 mm away from the fiber probe, was captured to measure the xenon arc lamp’s input spectrum. It should be noted that for this procedure, the light coupling between the xenon arc lamp and the input fiber was improved compared to our prior measurements, which necessitated a longer integration time. Interactance spectra were then calculated using Eq (1).

### 2.5 Establishing signatures from the skin and underlying tissues

**Fig 4:**
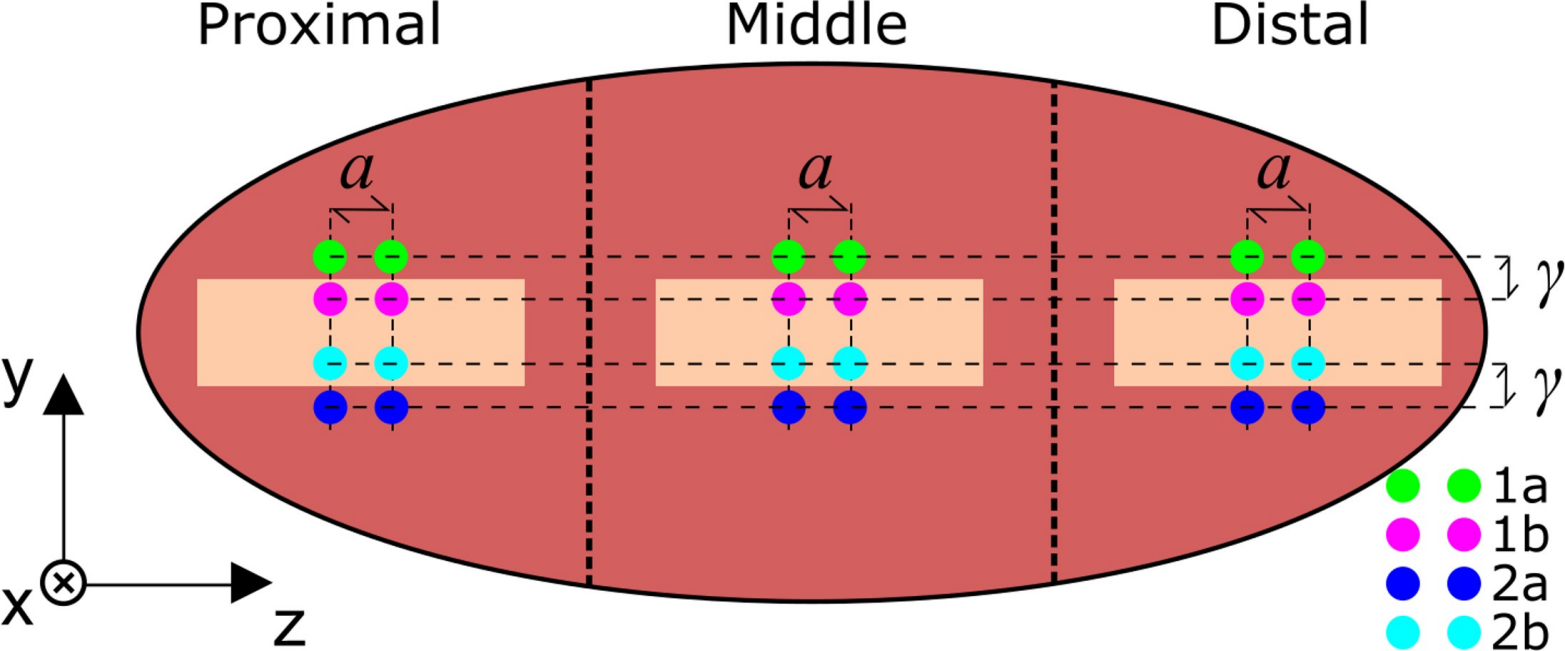
Sampling of full sweetpotatoes with skin removed. A patch of skin was removed from each of the three sections (proximal, middle, and distal). Four measurements were taken with the probes positioned at locations denoted 1a, 1b, 2a, and 2b corresponding to green, magenta, blue, and cyan, respectively. Note that 1a and 2b were measured with the skin in place and 1b and 2a were measured with the skin removed.

The batch containing 10 SPs, from the prior protocol, were also used to quantify the interactance spectrum’s variability from samples with and without the skin. Using a peeler, the skin was removed from one 10×25 mm spatial area within each of the proximal, middle, and distal regions from each of 5 randomly selected SPs. The probe was then used to acquire a total of four spectra in each region, as depicted in **Fig 4** where 1a and 2b were measured with the skin in place and 1b and 2a were measured with the skin removed. As before, the general 3-step interactance spectrum measurement procedure of section 2.3 was implemented here, where step (2) was modified to use an integration time of 250 ms due to the improved lamp coupling described previously in section 2.4. Additionally, pairs of spectra (pair 1 and 2) were collected such that the input and output fiber probes were close (*γ* < 5 mm) to the skin on (measurement *n*a) or skin off (measurement *n*b) boundary, where *n* is an integer denoting the measurement index. From these two measurements, the skin’s influence and variability can be separated from that of the underlying tissues by

$$I_{Skin}=\left(I_{Scattering,na}-I_{dark}\right)/\left(I_{Scattering,nb}-I_{dark}\right),$$ (3)

where *I*_*Scattering*_,_*n*a_ and *I*_*Scattering*_,_*n*b_ are the scattering spectra measured in both regions. Additionally, the variability of the underlying tissue can be studied across all samples after normalizing *I*_*Scattering*_,_*n*b_ to the xenon arc lamp’s spectrum using Eq (1).

### 2.6 Destructive ground-truthing of whole sweetpotatoes

After the spectra were measured, each SP sample from both batches was destructively measured by cutting it into slices with a thickness of 15±2.3 mm. Each slice was then photographed using a smartphone camera (a Samsung Galaxy S8 for the 10 sample batch and an Apple iPhone 9 for the 12 sample batch) mounted at a distance of 450 mm above the slice. The white spectralon tile was positioned in the field of view to enable color balance compensation, while a 25.4 mm diameter ring was used to enable spatial calibration. These images were used to visually quantify necrotic tissues within each of the 22 SP samples.

## 3.0 Experimental results

### 3.1 Pure healthy and necrotic tissues

**Fig 5:**
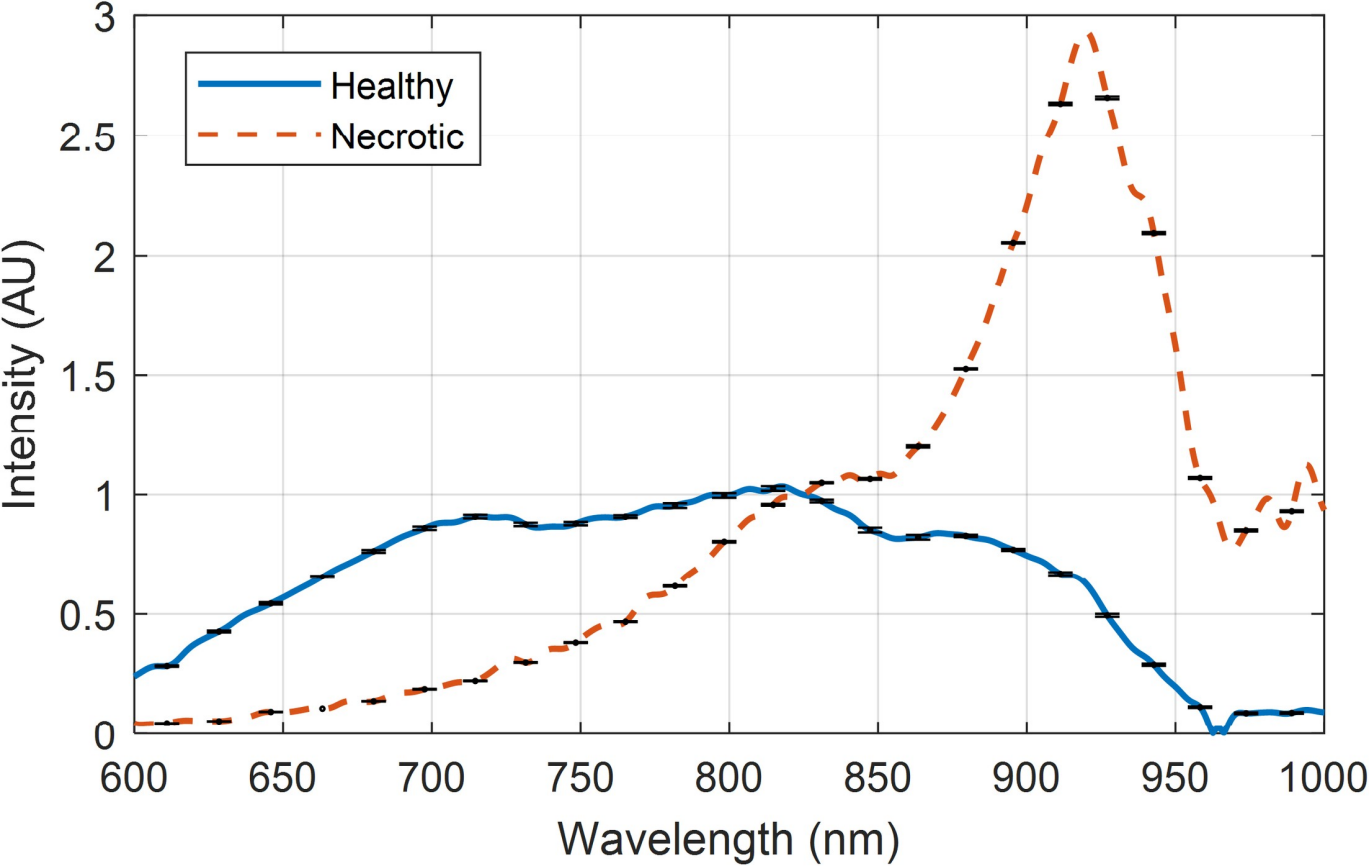
Pure tissue spectra. Normalized interactance spectra from the two tissue types. Error bars are based on the signal to noise ratio.

These normalized interactance spectra, from both pure healthy and necrotic tissues, are depicted in **Fig 5**. Generally, the presence of necrosis decreases the light’s ability to scatter and transmit within the tissue at shorter wavelengths spanning 600–900 nm, relative to wavelengths longer than 900 nm. Conversely, healthy tissue transmits shorter wavelengths with higher relative efficiency from 600–900 nm. Spectra have been normalized at a value of 825 nm to ease interpretation.

### 3.2 Internal necrosis measurements through the skin

**Fig 6:**
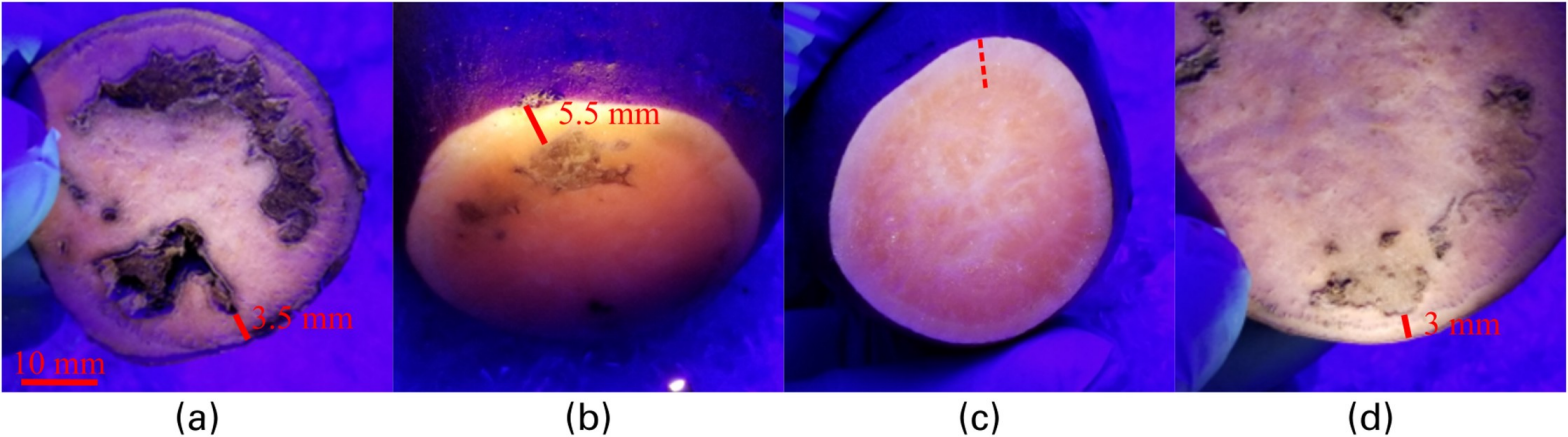
Pictures of healthy and necrotic tissues at different depths. (a) necrotic tissue observed at the proximal end of a sweetpotato at a depth of 3.5±0.5 mm; (b) necrotic tissue at a depth of 5.5±0.5 mm towards the middle of the SP, (c) mid-section of a healthy sweetpotato; and (d) mid-section of a necrotic sweetpotato with necrosis at a depth of 3.0±0.5 mm.

Healthy and necrotic tissue were sampled from the *Covington* cultivar. Healthy and necrotic tissue were sampled from Covington sweetpotato tubers. Representative red, green, and blue (RGB) pictures are depicted in **Fig 6** for necrotic tissues observed at different depths (a, b, and d) and for healthy tissue (c).

**Fig 7:**
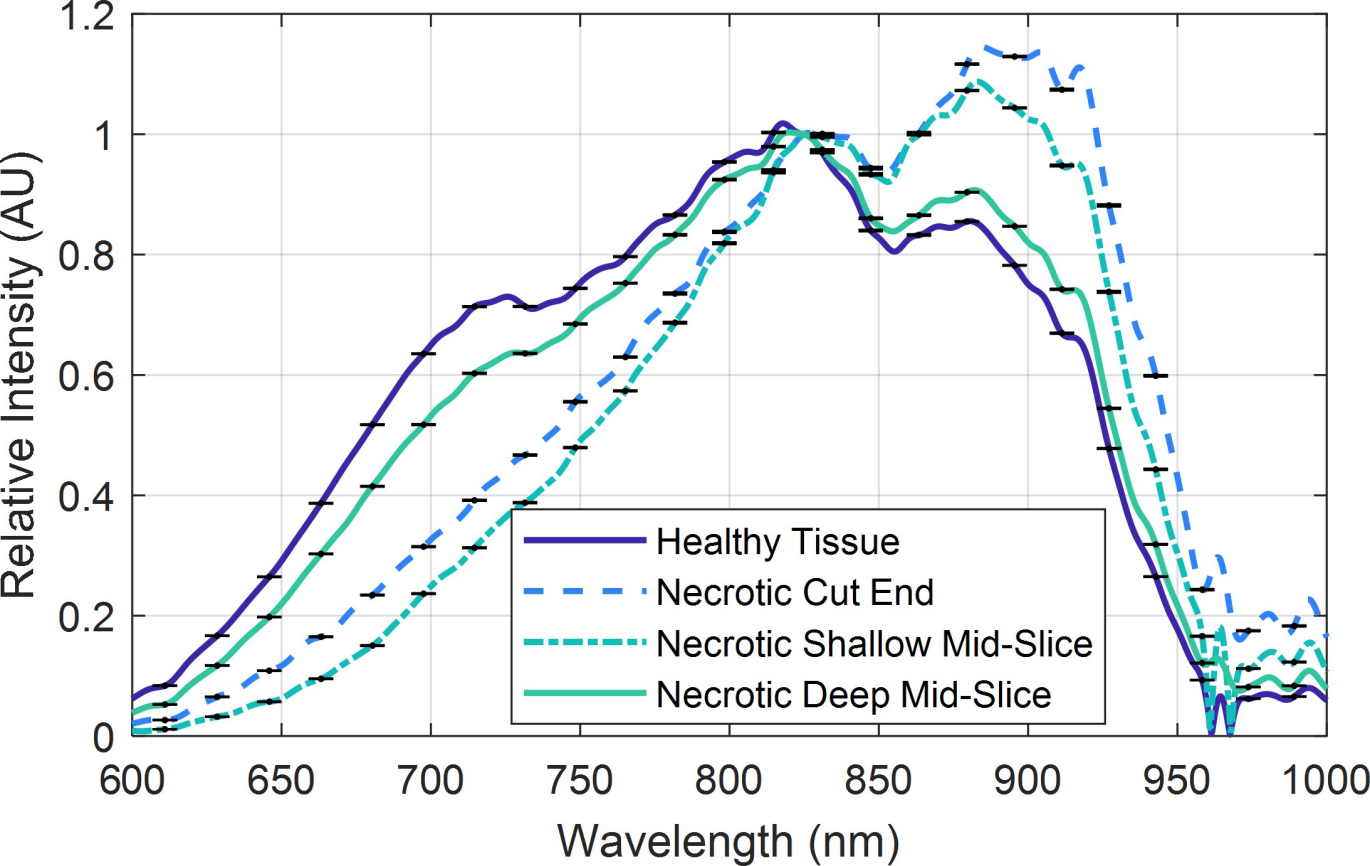
Measured interactance spectra. Data were acquired from healthy and necrotic tissues at the different depths as reported in Fig 6. Error bars are based on the signal to noise ratio.

One interactance spectrum was measured at each of the locations indicated in **Fig 6(A)–6(D)** and are presented in **Fig 7**. Spectra have been normalized, using Eq (2), to ease interpretation. As the necrotic tissue’s depth decreases, there is a tendency in the spectra to experience an increase in relative intensity for wavelengths spanning 850–950 nm. Conversely, there is generally a decrease in relative intensity for wavelengths spanning 600–800 nm. Furthermore, the deeper necrotic tissue at 5.5 mm is similar to the healthy tissue.

### 3.3 Measuring the spectral variability among sweetpotatoes

**Fig 8:**
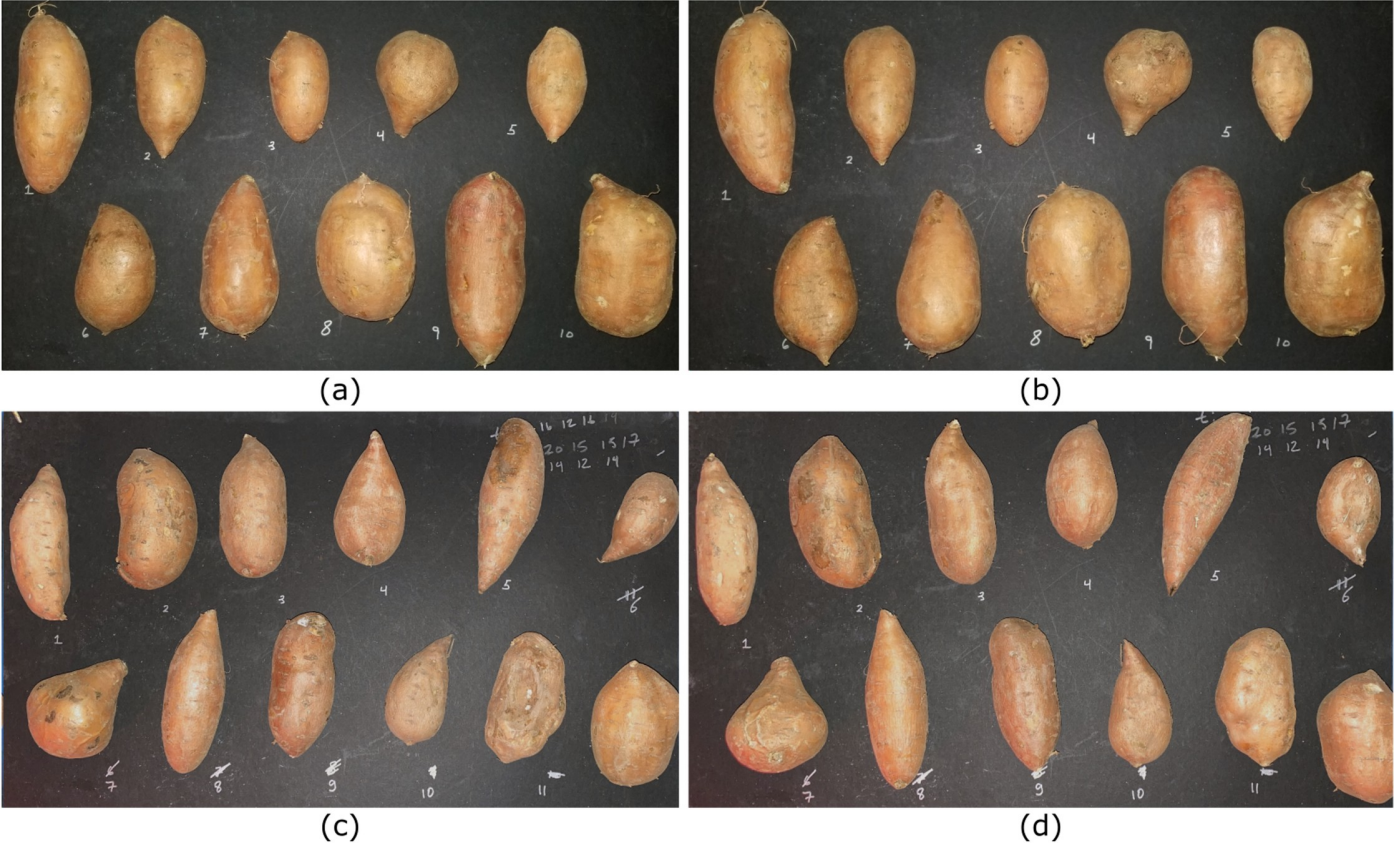
RGB imagery of the two batches of SPs that were measured. (a) top and (b) bottom side of the first batch and (c) top and (d) bottom side of the second batch.

The RGB imagery, acquired of the first and second batches of 10 and 12 SPs, are depicted in **Fig 8(A), 8(B)** and **8(C), 8(D)**, respectively.

**Fig 9:**
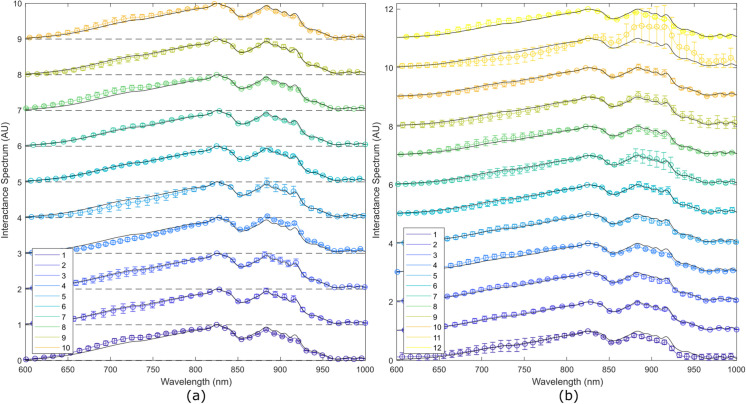
Interactance spectra from the 22 samples. (a) First batch containing 10 samples; and (b) second batch containing 12 samples.

All 12 interactance spectra, measured from each SP, were used to calculate the mean and standard deviation. These results are depicted in **Fig 9(A)** and **9(B)** for the first and second batches, respectively. Note that error bars in these figures represent one standard deviation from the mean. Each SP was numbered by an index 1–10 for the first batch of 10 SPs and 1–12 for the second batch of 12 SPs. The spectral variability among the SPs is largest around 750 nm and 900 nm due to the presence of necrotic tissue in the samples.

**Fig 10:**
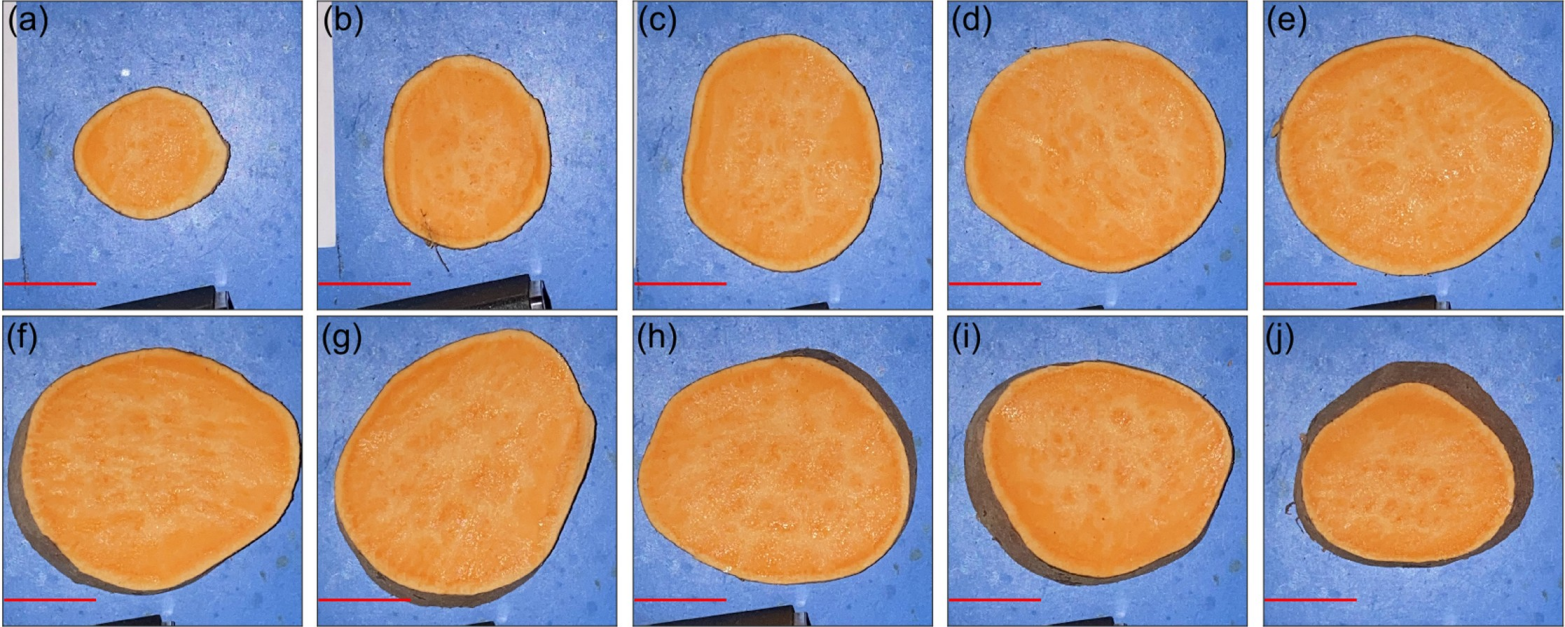
Healthy tissue RGB cross-sectional images of SP 3 from batch 2. Slices are depicted from the top to bottom (with reference to the orientation in **Fig 8**) for panels (a-j). The red scale bar in each panel represents a distance of 25 mm.

Ground truth images were also collected from each SP using the protocols detailed in section 2.6. Cross-sectional images of SP 3 from batch 2 are depicted in **Fig 10(A)–10(J)**, which serves as a healthy representative sample.

**Fig 11:**
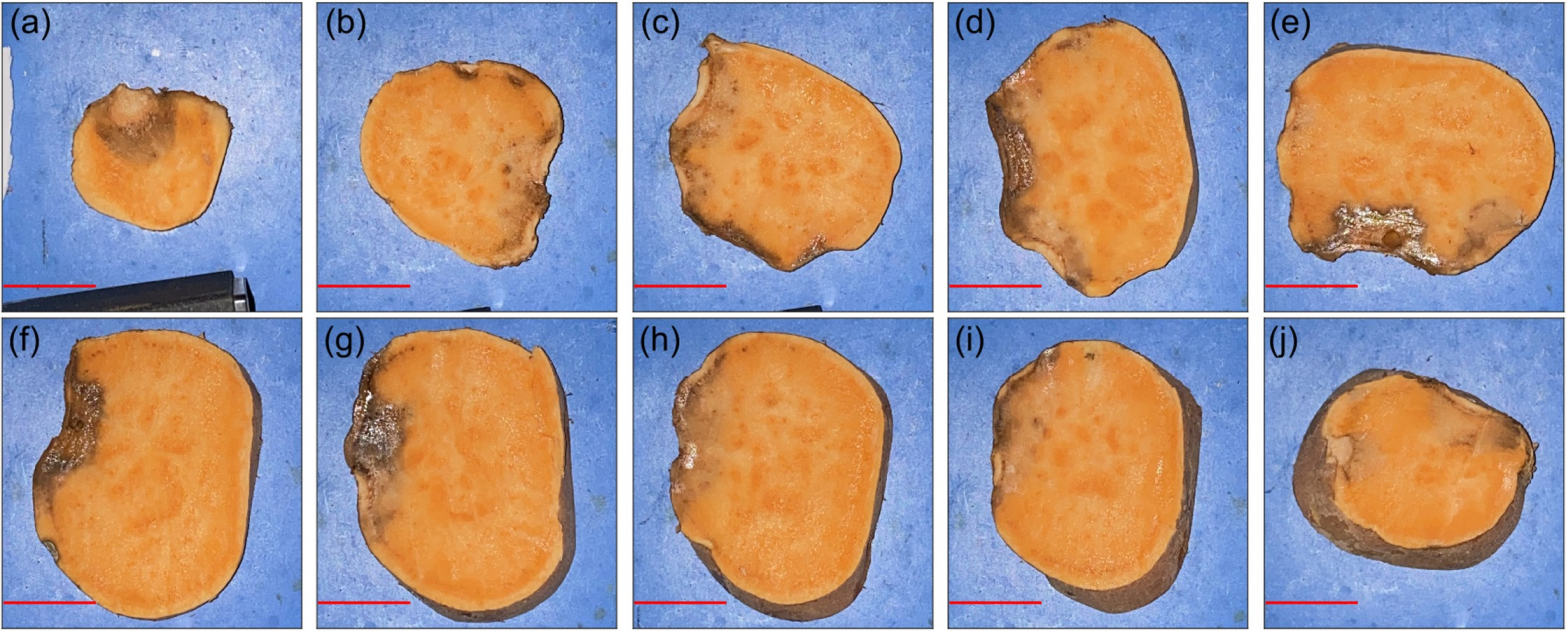
Necrotic tissue RGB cross-sectional images of SP 11 from batch 2. Slices are depicted from the top to bottom (with reference to the orientation in **Fig 8**) for panels (a-j). The red scale bar in each panel represents a distance of 25 mm.

Of all the measured SPs, the one containing the most significant amount of necrotic tissue is SP 11 from batch 2, depicted in **Fig 11(A)–11(J)**.

Additional data are provided in **S**1**A–S**1**J Fig** in S1 Fig for batch 1 and **S**1**K–S**1**G Fig** in S1 Fig for batch 2. From these ground truth data, necrotic tissue was also present on SP 9 and SP 7 in batch 2, which presented itself as an increased standard deviation around 900 nm in the interactance spectra.

### 3.4 Measurements of skin and underlying tissues

**Fig 12:**
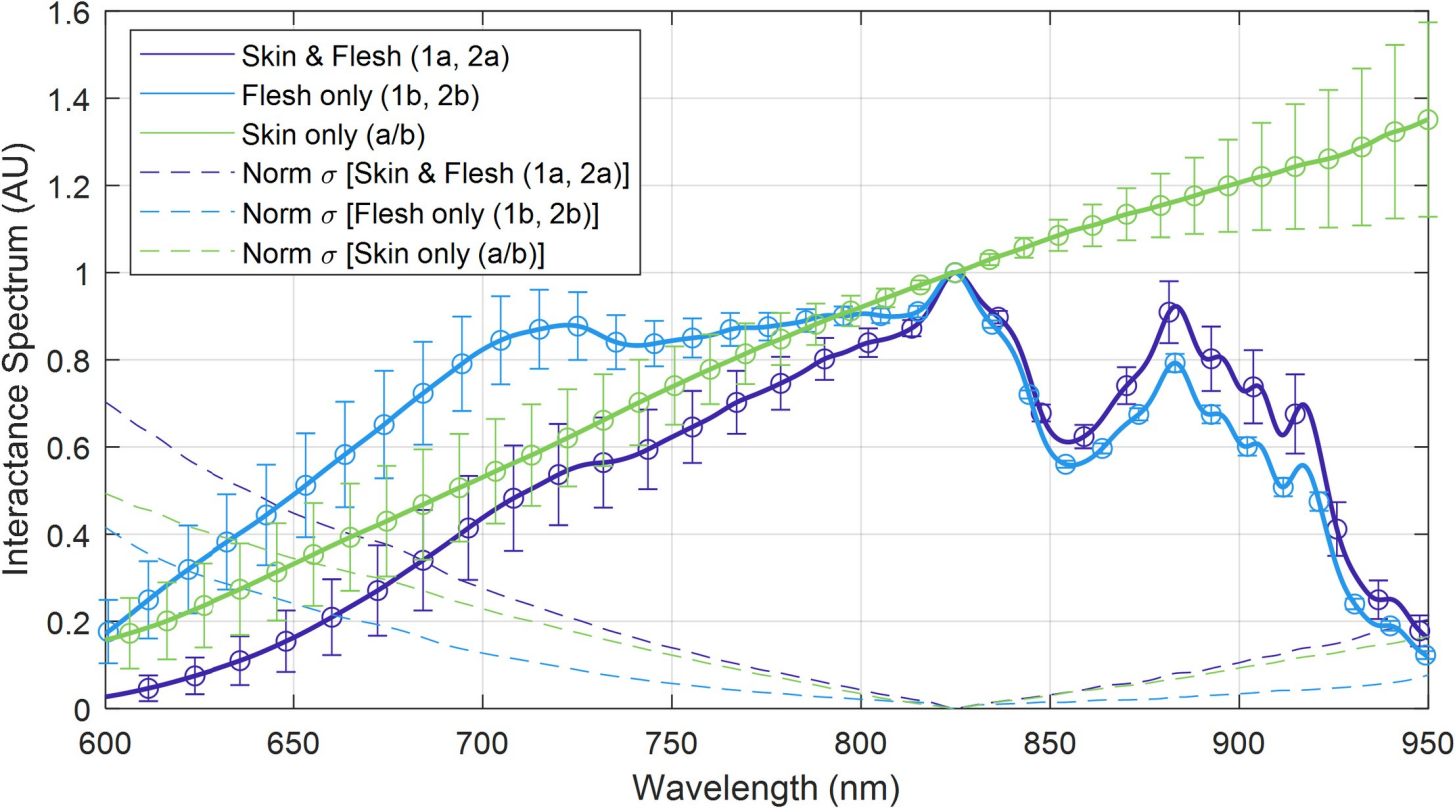
Interactance spectra of the skin and flesh. Recall that 1a and 2a were taken through the skin while 1b and 2b were taken with the skin removed.

A total of 5 SPs were randomly selected, using a random number generator in *Matlab*, from the first batch of 10 SPs corresponding to SP indices 1, 2, 4, 7, and 9. Interactance spectra were measured using the procedure of section 2.5 with processing and normalization per section 2.2. All spectra from a given SP were averaged, and their standard deviation was calculated. A view of the data are presented in **Fig 12**, where the mean value was calculated across all 30 measurements (3 sites, 5 sweetpotato samples, 2 replications) for each measurement site location per **Fig 4**.

Also presented are the normalized standard deviations for each curve. The skin’s influence is also presented as the normalization of the 1a, 2a measurements to the 1b, 2b measurements, respectively, as calculated by Eq (3).

## 4.0 Optical modeling and simulations

**Fig 13:**
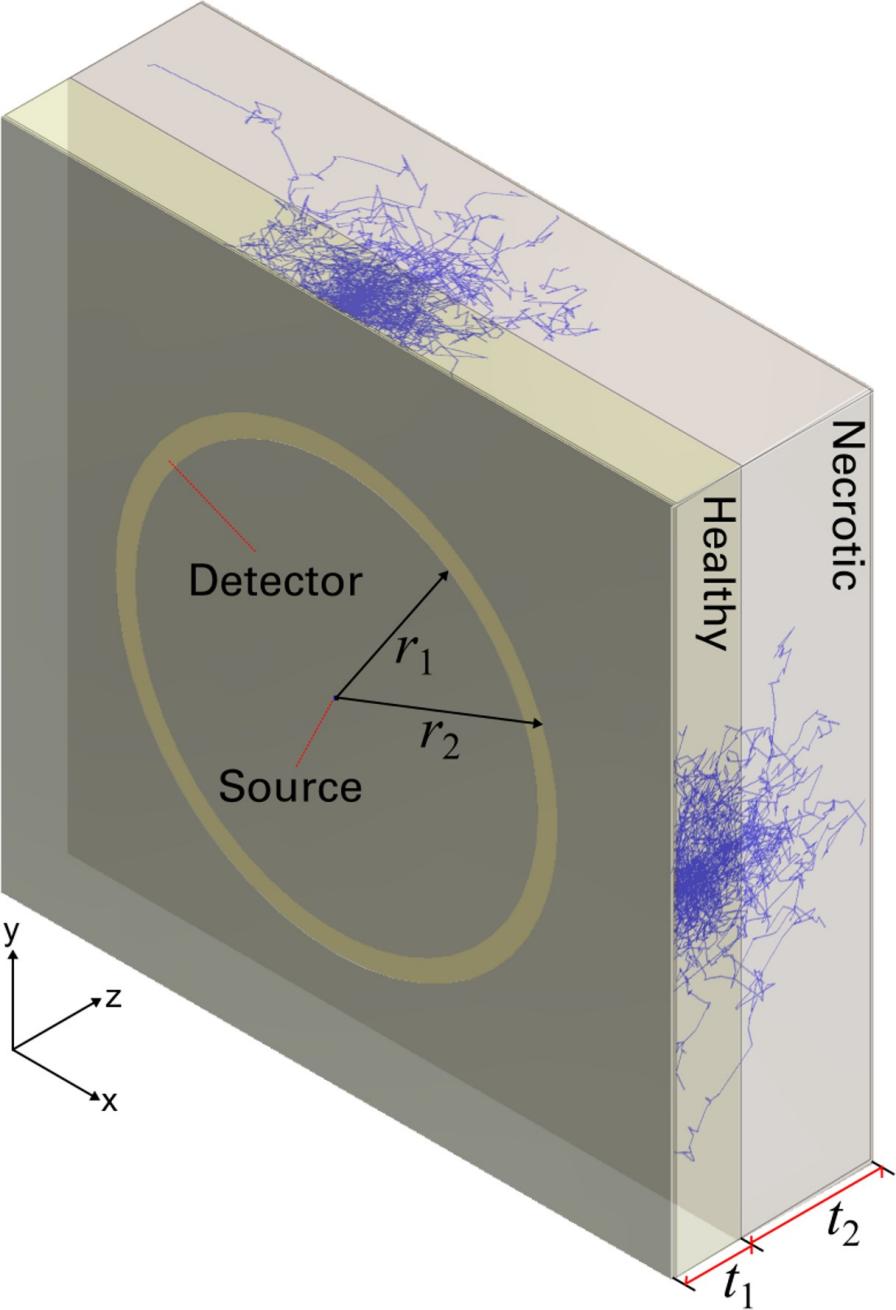
Zemax scattering simulation. Healthy and necrotic tissues are configured with thicknesses of *t*_1_ and *t*_2_, respectively. An annular detector with an active area defined by inner and outer radii *r*_1_ and *r*_2_, respectively.

A major goal of our future work will be to develop a high throughput sensor system capable of quantifying IN, and other indicators of necrosis in high-speed sorting and packing facilities. To facilitate an online sensor’s optical design, a Zemax scattering model was configured to simulate the signatures presented previously in **Fig 7**. Furthermore, we used this model to quantify the likely depth limit of the measurement technique on SPs. The simulation setup is depicted in **Fig 13**. It consists of a collimated point source located just outside (0.1 mm) of a rectangular volume that defines the healthy tissue. The healthy tissue has a thickness of *t*_1_ and sits above another rectangular volume that defines the necrotic tissue with thickness *t*_2_. Light rays undergo multiple scattering within these volumes, some of which are re-directed towards an annular detector area. This detector lies within the same plane as the source and has an outer radius *r*_1_ and inner radius *r*_2_. This configuration enables light to be measured at various simulated distances away from the source in the center. However, in our simulations, we focus only on modeling for a distance of *a* = 8.5 mm, which corresponds to the distance between the source and detector in our experimental trials.

**Fig 14:**
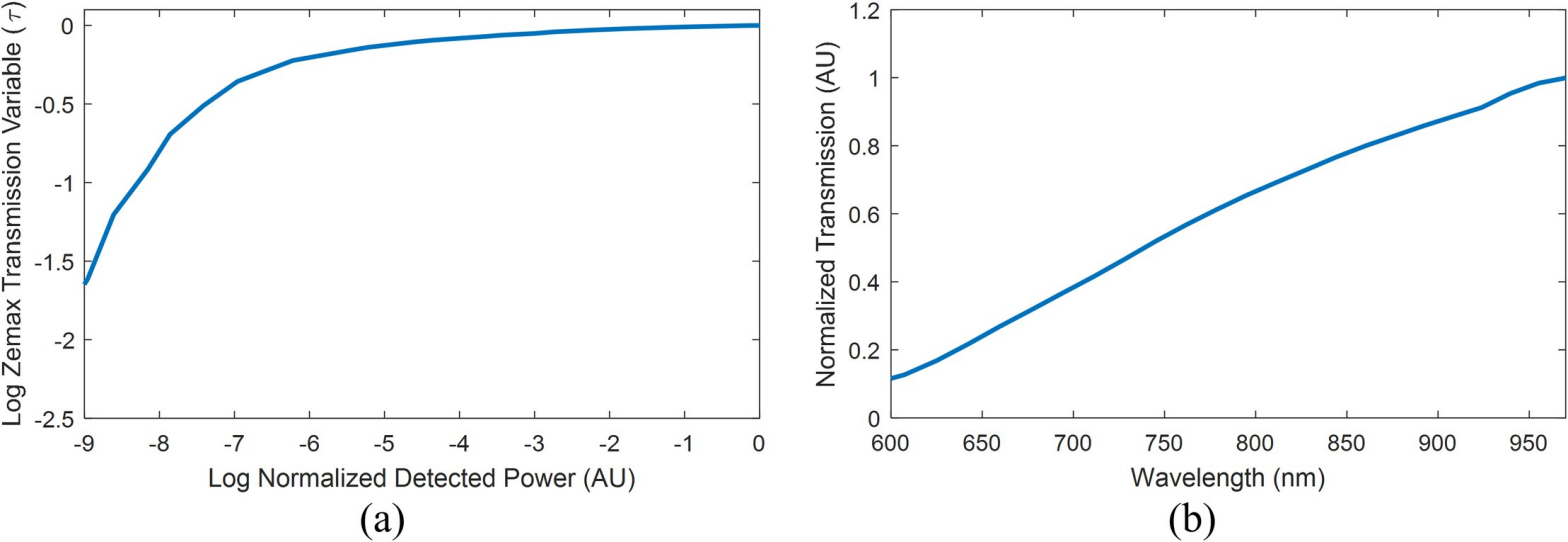
Data used in the model for fitting. (a) Graphical solution for τ versus the normalized detected power; (b) Mean skin transmission spectrum (double-pass) from **Fig 12**(c).

Using the built-in scattering function bulk_samp_1.dll, a bulk scattering surface has two parameters: the mean scattering free path *l* and the transmission of the scattering particles *τ*. In this model, *τ* defines the fractional energy that is transmitted upon each scattering event. To simplify our measurements, we assumed that the light scattering properties of sweetpotatoes are similar to that of white potatoes, but with different absorption characteristics caused by the increased pigmentation in SPs. This assumption enabled us to set *l* to a constant versus wavelength of 0.65 mm^-1^ [32]. For *r*_1_ = 8 mm and *r*_2_ = 9 mm, the detected intensity was simulated for transmission values spanning 0.3 to 0.999, given a thick scattering layer such that *t*_1_ = 50 mm. These results were used to create a graphical solution of *τ* as a function of the desired normalized detected power, depicted in **Fig 14(A)**. By interpolating this function, *τ* can be calculated at each wavelength by inputting the normalized interactance spectra. Finally, a measurement of the skin spectrum was captured per **Fig 14(B)** such that the simulated data from Zemax could have a double-pass of the skin layer applied to them.

**Fig 15:**
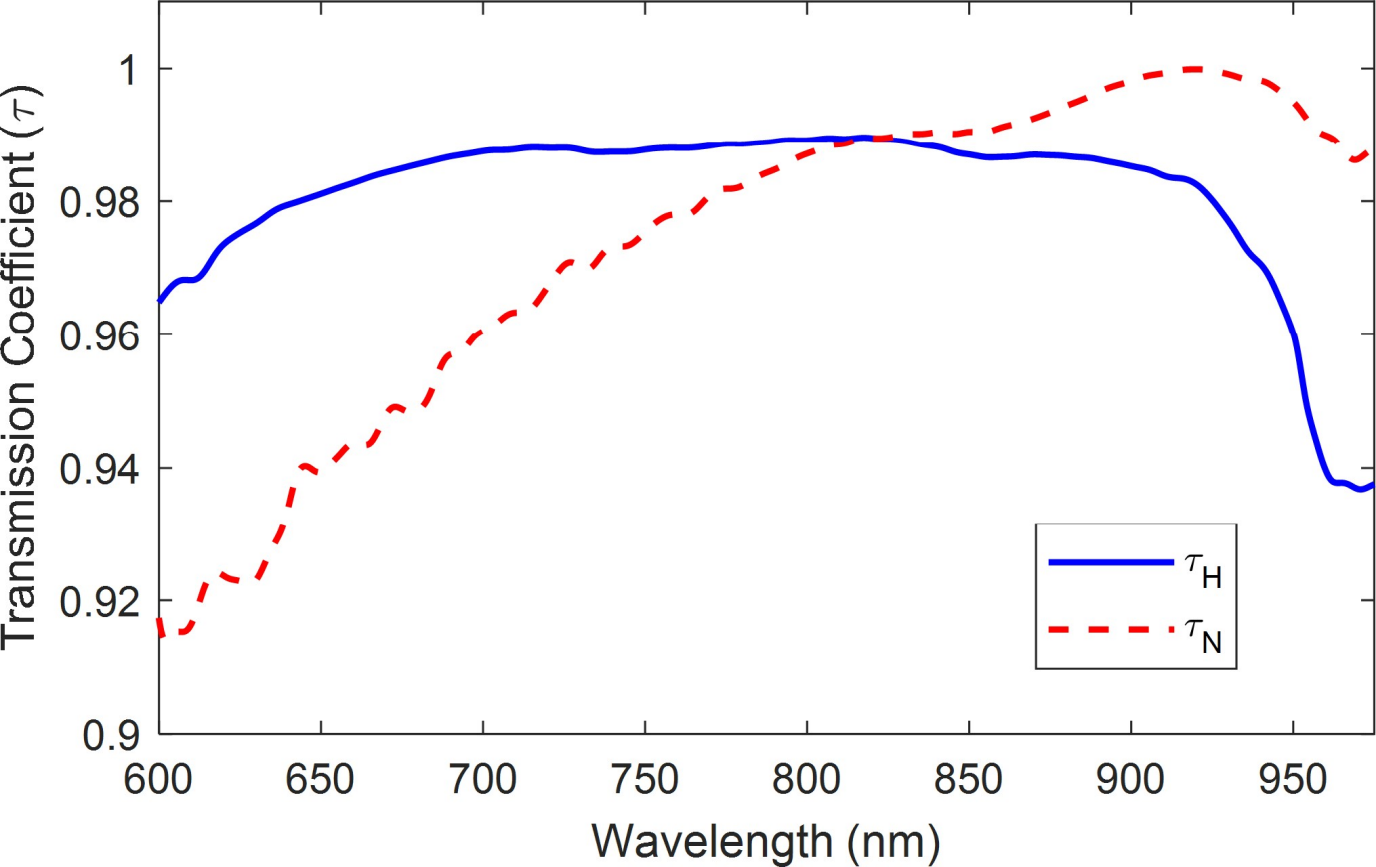
Bulk scattering transmission coefficients for pure healthy and necrotic tissue.

The model was configured using the pure-sample interactance spectra depicted in **Fig 5**. Since interactance spectra are a relative measure of scattering absorption for a given sensor platform (in this case, probe geometry), the pure tissue spectra were normalized to the maximum of the necrotic tissue maxima of 2.932. These spectra were then converted using the data of **Fig 14(A)** using cubic interpolation. This produced the coefficients *τ*_*H*_ and *τ*_*N*_ for the healthy and necrotic tissue, respectively, depicted in **Fig 15**. These values were input into the *Zemax* model, using a macro, to perform the ray tracing at each wavelength. Outputs were written to a comma-separated value file, which was imported into *Matlab* before multiplying the simulated spectra by the skin’s transmission spectrum per **Fig 14(B)** and normalizing the spectrum per Eq (2).

To investigate the model’s ability to predict the trends in the measured interactance spectra, simulations were performed with *r*_1_ = 8 mm and *r*_2_ = 9 mm. Healthy tissue thicknesses spanning *t*_1_ = 1 mm to *t*_1_ = 8 mm, in 1 mm increments, were used above a thick necrotic layer of *t*_2_ = 50 mm.

**Fig 16:**
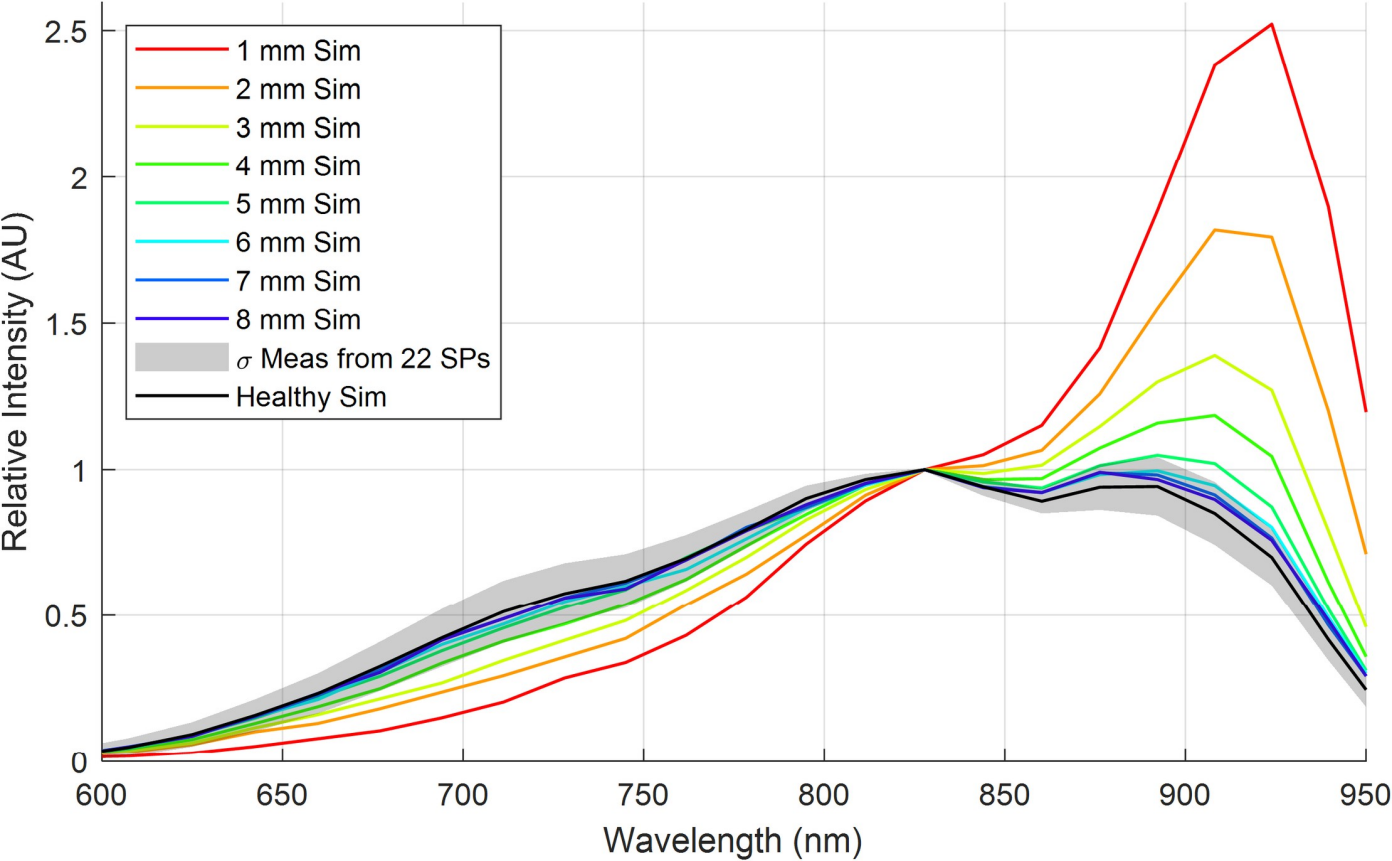
Simulation results for necrotic tissues at depths *t*_1_ spanning 1 to 8 mm in 1 mm increments (red through violet sold lines, respectively). Error bars from the measured data are included on the simulated healthy spectrum (black solid line) out to one standard deviation.

The results from this scattering simulation are depicted in **Fig 16** for each thickness *t*_1_. Also included is the simulated measurement when *t*_1_ = 50 mm of healthy tissue, which represents an SP with no internal necrotic tissue. Superimposed onto this line is the standard deviation of the interactance spectra, calculated from the measured data across all 22 samples that were quantified in our random sampling per section 3.3. This provides a relative sense of how the skin’s variability might impact the maximum reliable depth to which the given probe geometry could quantify the necrotic tissue. As previously observed in **Fig 7**, there is a decrease in the relative scattering efficiency for wavelengths less than 825 nm and an increase in efficiency for wavelengths greater than 825 nm.

**Fig 17:**
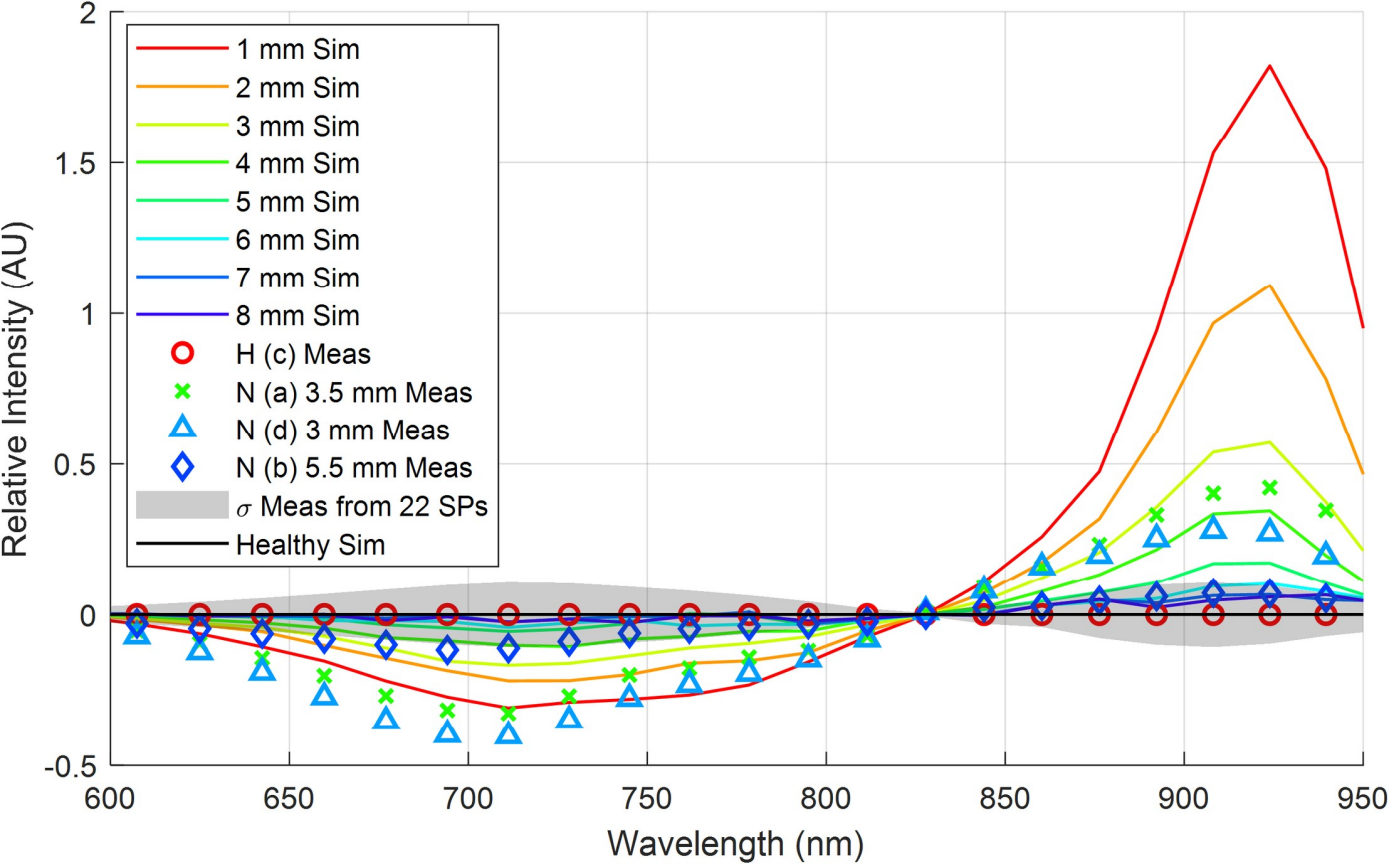
Simulation and measured results after subtracting the healthy reference spectra. Simulation results are provided for necrotic tissues at depths *t*_1_ spanning 1 to 8 mm in 1 mm increments (red through violet sold lines, respectively). Healthy and necrotic tissues.

Finally, the simulation results were compared to the measured data, presented previously in **Fig 7**, by taking the normalized measured or theoretical interactance spectra and subtracting the measured or theoretical healthy reference spectrum, respectively. These results are depicted in **Fig 17**. Generally, the magnitudes of the experimental and theoretical spectral signatures are similar for wavelengths greater than 825 nm, while there is a more significant error for wavelengths shorter than 825 nm.

## 5.0 Discussion

As hypothesized, results indicate that the interactance method enables the detection of sub-dermal necrosis in the *Covington* sweetpotato cultivar. Furthermore, the trends in the optical model simulated the trends in the measured data, as depicted in **Figs 16** and **17**. The measured data and the simulated results indicate that tissue necrosis may be reliably detected to a depth of approximately 5 mm, beyond which the signature peak around 925 nm starts to become masked by the expected variability in the healthy tissue and skin spectra presented in **Fig 12**. Internal necrosis begins at the proximal end and propagates through the sweetpotato tissue towards the distal end. In this region of the SP, the necrotic tissue lies close to the surface (around 1–3 mm deep). We expect that imaging the ends of the SP will best enable the detection of IN when using interactance spectroscopy. Furthermore, in our random testing presented in section 3.3, an SP containing necrotic tissue was quantified in which tissue necrosis was caused by Fusarium dry rot per the ground-truth measurements depicted in **Fig 11**. Despite a different cause, necrotic tissue presented a similar trend in the spectrum in which the interactance spectra at 925 nm had an increased scattering efficiency relative to 825 nm.

One confounding factor for interactance spectral measurements includes the skin, which the photons must transmit through twice. The skin introduces variability depending on which spatial location the interactance spectra are collected. From the normalized standard deviation of the skin’s and flesh’s variability per **Fig 12**, it is evident that:

1. The skin is highly variable both above and below 825 nm, meaning it can increase or decrease the slope (intensity per nm) that is induced by the presence of necrotic tissue;
2. For wavelengths longer than 825 nm, the skin is the primary cause of variability, whereas the underlying flesh’s interactance spectra are stable.
3. For wavelengths less than 825 nm, the skin is still the primary cause of variability, but the underlying flesh contributes more significantly than for longer wavelengths.

The variability in the skin’s calculated transmission spectra may also explain some of the differences between the measured and simulated results. Comparing the 3 mm deep tissue measurements to the simulated data for a 3 mm depth in **Fig 17** yields a peak error of approximately 20% for wavelengths less than 825 nm and a peak error of approximately 10% for wavelengths greater than 825 nm. The higher error at shorter wavelengths supports the likelihood that the skin’s spatial variability is impacting the accuracy. From these observations, it is envisioned that the detection of necrotic tissue may be more consistent for wavelengths longer than 825 nm.

A strategy that could be used to correct for the skin’s influence may be to quantify the reflectance spectrum of the skin before, or in tandem, with the interactance spectrum’s measurement. Further work will need to be conducted to determine how well correlated these reflection spectra are in comparison to the interactance spectra and the skin’s transmission spectrum that was calculated in **Fig 12**. We expect that results should be translatable to other white- and yellow-fleshed SPs; however, it is unlikely they would translate to more exotic varieties, such as purple-fleshed SPs. In this case, further measurements would be required to re-calibrate the models to the different pigments’ absorption properties.

## 6.0 Conclusions

In this study, we demonstrated that interactance spectra have the potential to detect internal necrosis in sweetpotatoes non-destructively. Measurements indicate that necrotic tissue can be detected by a relative increase at 925 nm compared to 725 nm when normalized to 825 nm. Detection is reliable to a depth of approximately 5 mm with the given probe spacing of 8.5 mm. A *Zemax* simulation, created from our measurements of healthy and necrotic tissues was able to replicate the trends found in the measured data. Finally, we demonstrated that a significant confounding factor to the measurement’s consistency is the skin’s spatial variability. While we expect that a normalization method, in which the skin’s reflectivity is used to substitute for the skin’s unknown transmission, further research is required to demonstrate its efficacy and repeatability. However, it is anticipated that the interactance spectra have a high likelihood of measuring sub-surface defects in either handheld quality control or high throughput scanning, sorting, and packing applications. Our future work will be to resolve issues with the skin spectra and to leverage our *Zemax* model to develop a high throughput scanning sensor capable of quantifying necrotic tissues in packing and sorting facilities.

## Supporting information

S1 Fig (DOCX)
