# Supplementary material for: Internal defect scanning of sweetpotatoes using interactance spectroscopy
Source: PLoS One. 2021 Feb 9;16(2):e0246872. doi: 10.1371/journal.pone.0246872 (PMC7872240; doi:10.1371/journal.pone.0246872)
Supplement: S1 Fig — (DOCX) [file pone.0246872.s001.docx]

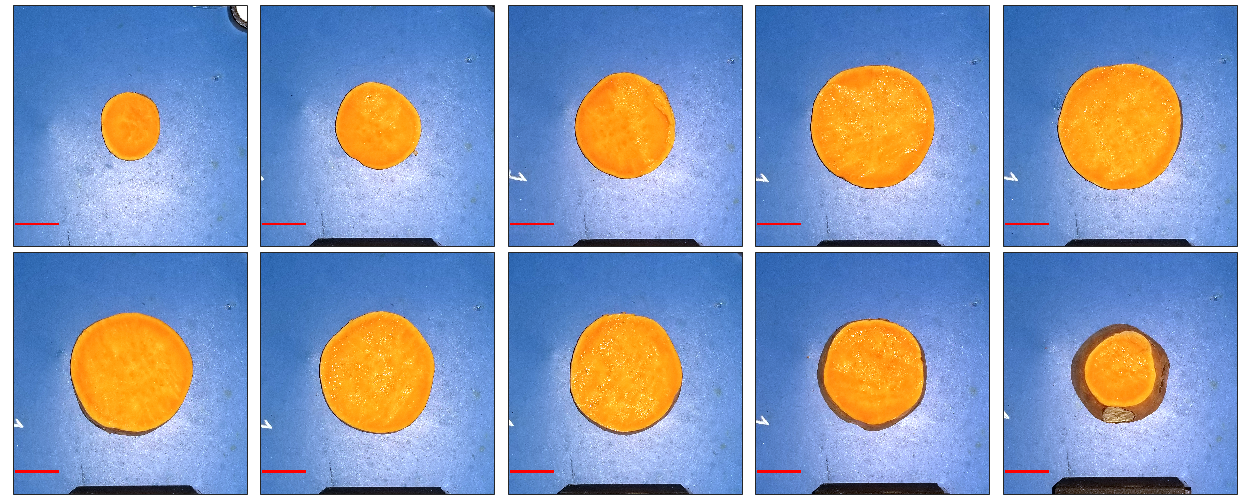


**S1A Fig. RGB Imagery of SP1 in Batch 1.** Red scale bar indicates 25 mm length. Each panel represents approximately 15±2.3 mm of thickness.


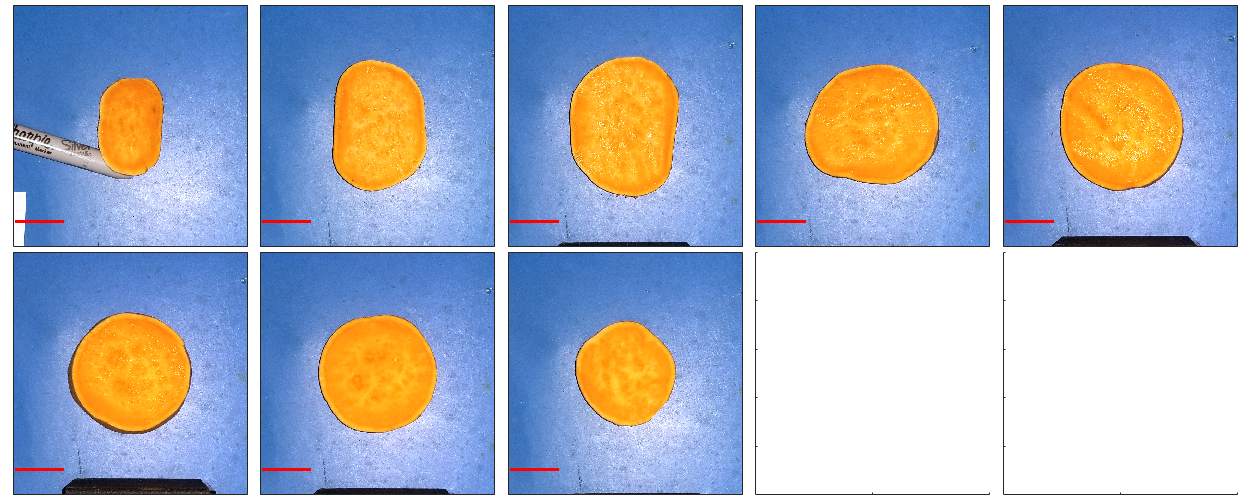


**S1B Fig. RGB Imagery of SP2 in Batch 1.** Red scale bar indicates 25 mm length. Each panel represents approximately 15±2.3 mm of thickness.


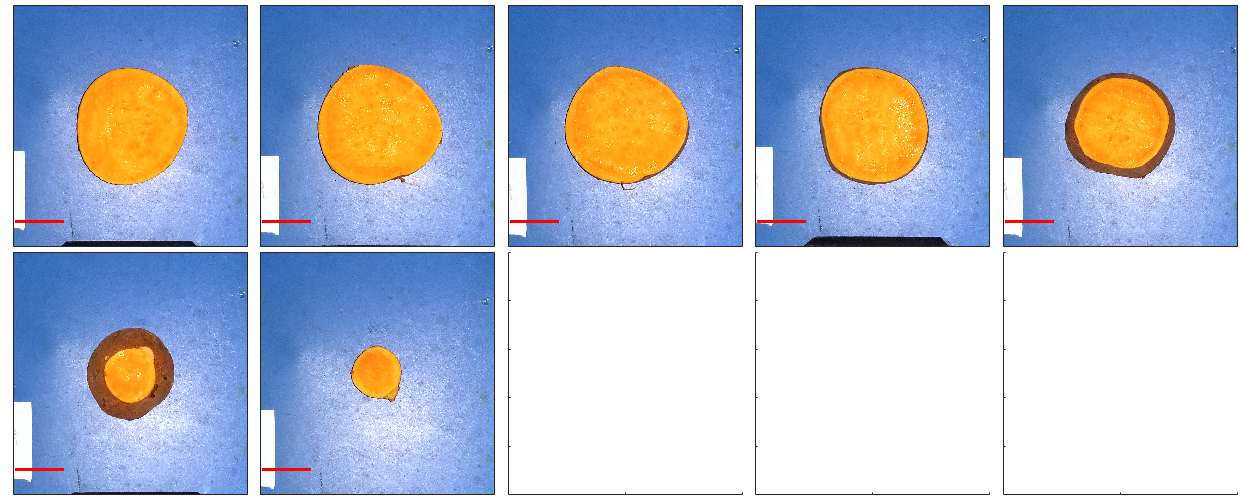


**S1C Fig. RGB Imagery of SP3 in Batch 1.** Red scale bar indicates 25 mm length. Each panel represents approximately 15±2.3 mm of thickness.


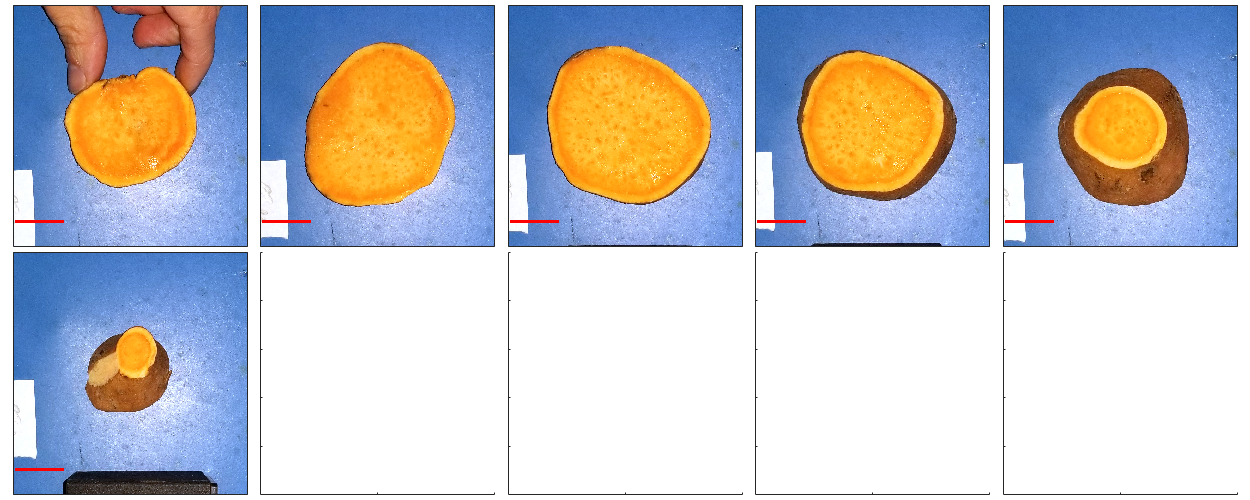


**S1D Fig. RGB Imagery of SP4 in Batch 1.** Red scale bar indicates 25 mm length. Each panel represents approximately 15±2.3 mm of thickness.


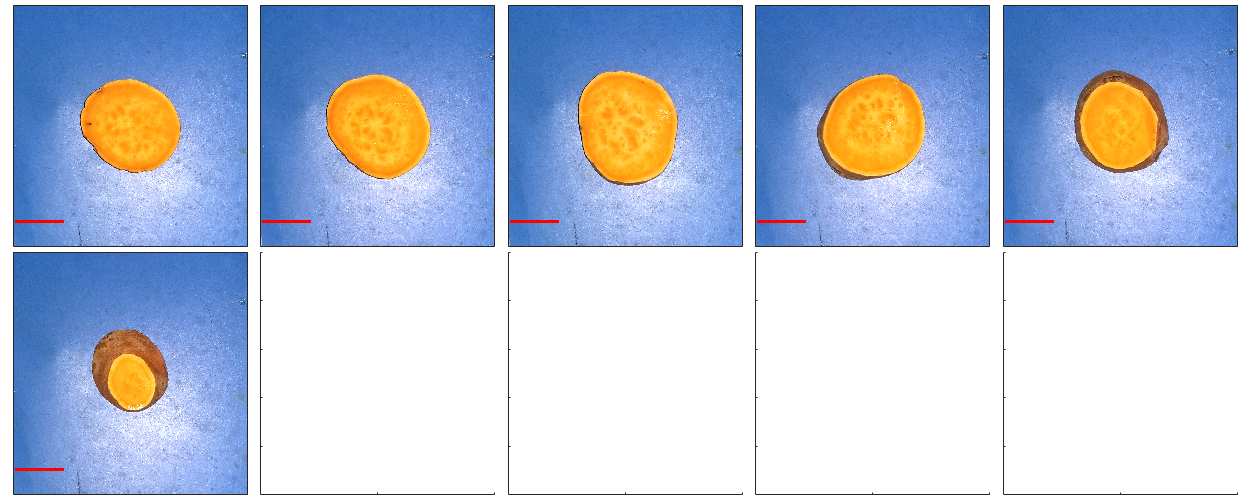


**S1E Fig. RGB Imagery of SP5 in Batch 1.** Red scale bar indicates 25 mm length. Each panel represents approximately 15±2.3 mm of thickness.


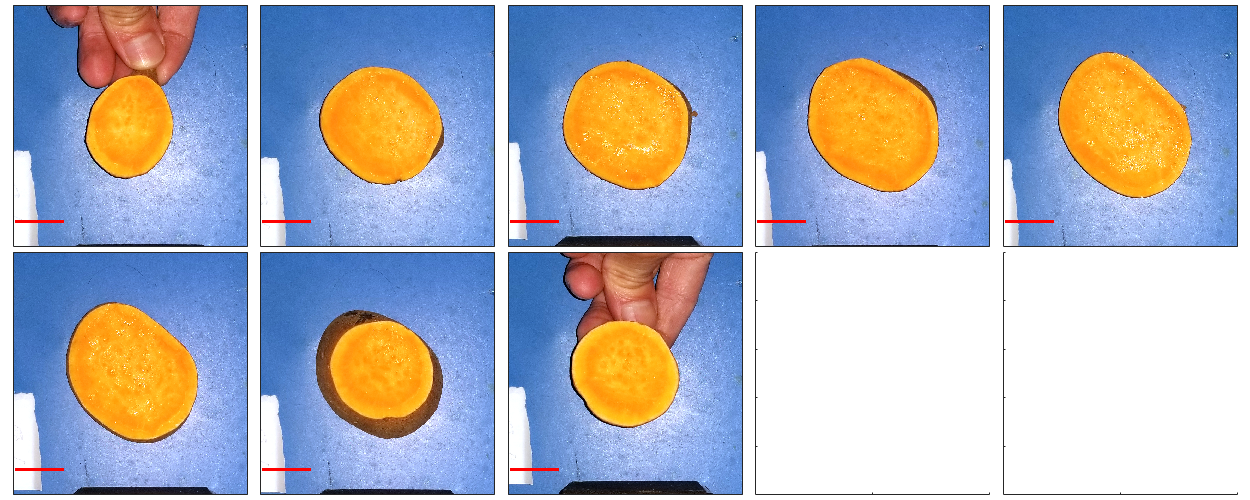


**S1F Fig. RGB Imagery of SP6 in Batch 1.** Red scale bar indicates 25 mm length. Each panel represents approximately 15±2.3 mm of thickness.


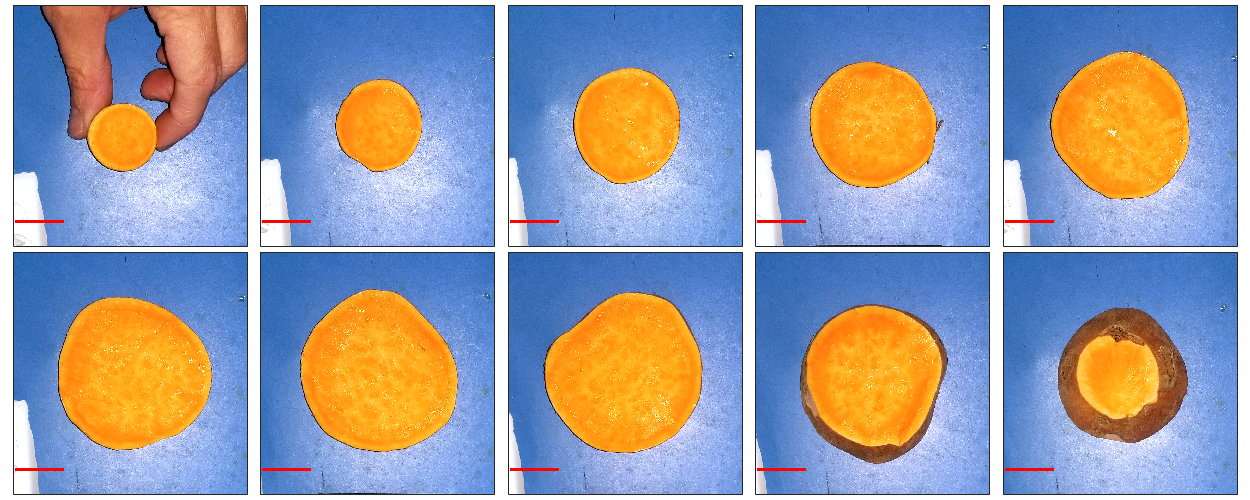


**S1G Fig. RGB Imagery of SP7 in Batch 1.** Red scale bar indicates 25 mm length. Each panel represents approximately 15±2.3 mm of thickness.


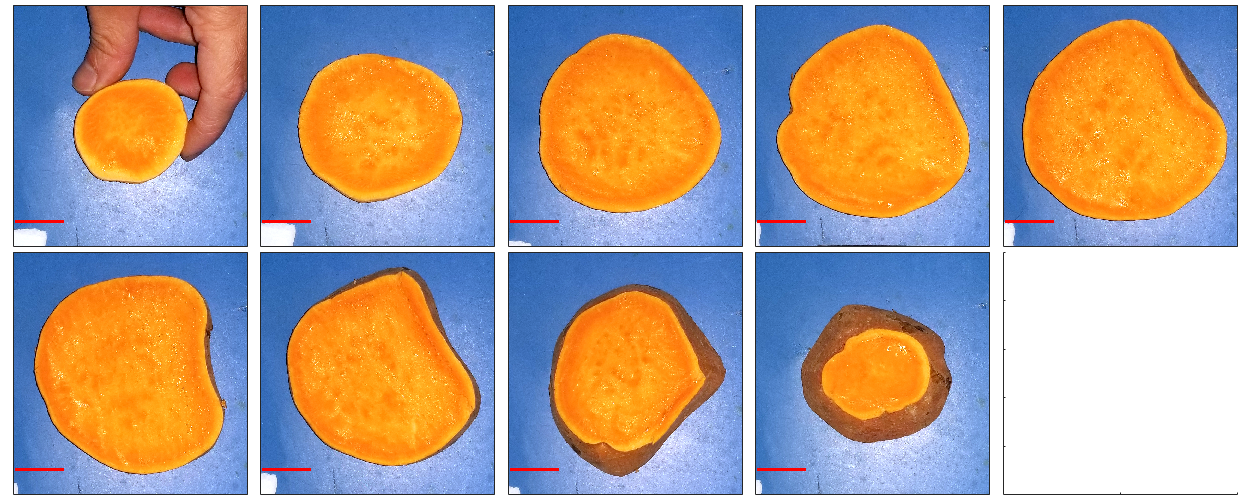


**S1H Fig. RGB Imagery of SP8 in Batch 1.** Red scale bar indicates 25 mm length. Each panel represents approximately 15±2.3 mm of thickness.


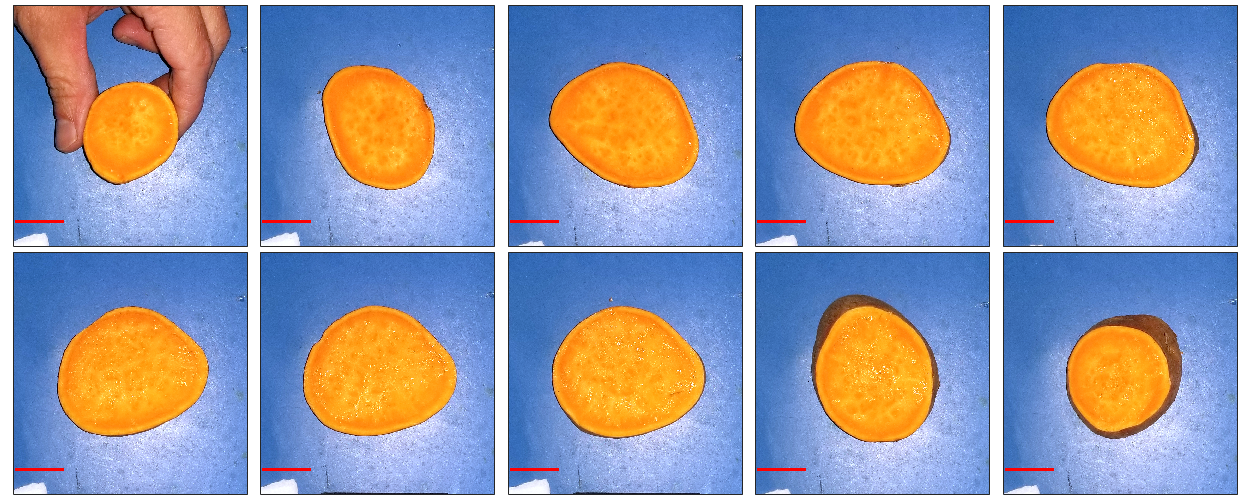


**S1I Fig. RGB Imagery of SP9 in Batch 1.** Red scale bar indicates 25 mm length. Each panel represents approximately 15±2.3 mm of thickness.


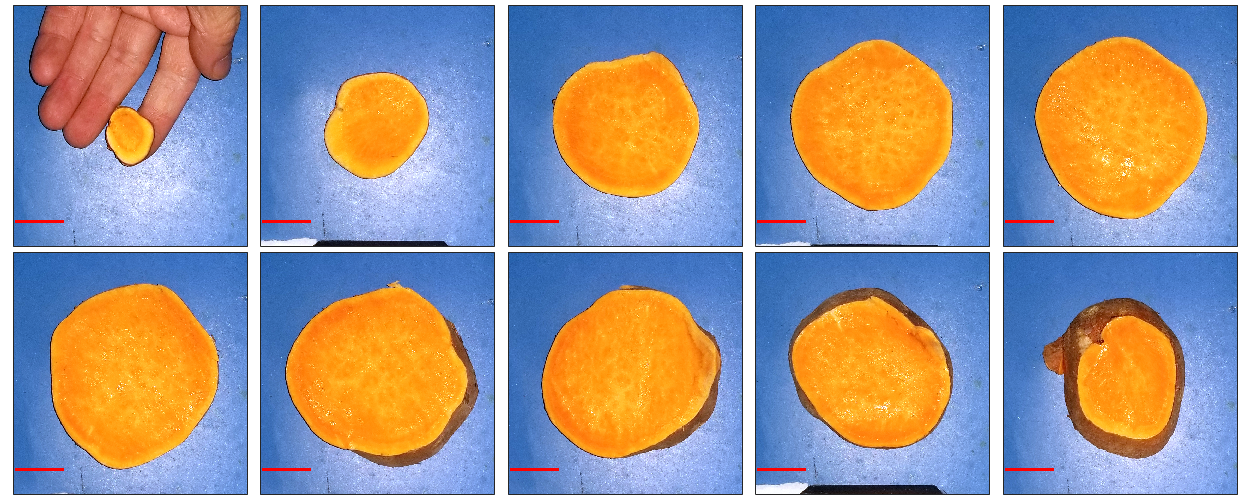


**S1J Fig. RGB Imagery of SP10 in Batch 1.** Red scale bar indicates 25 mm length. Each panel represents approximately 15±2.3 mm of thickness.


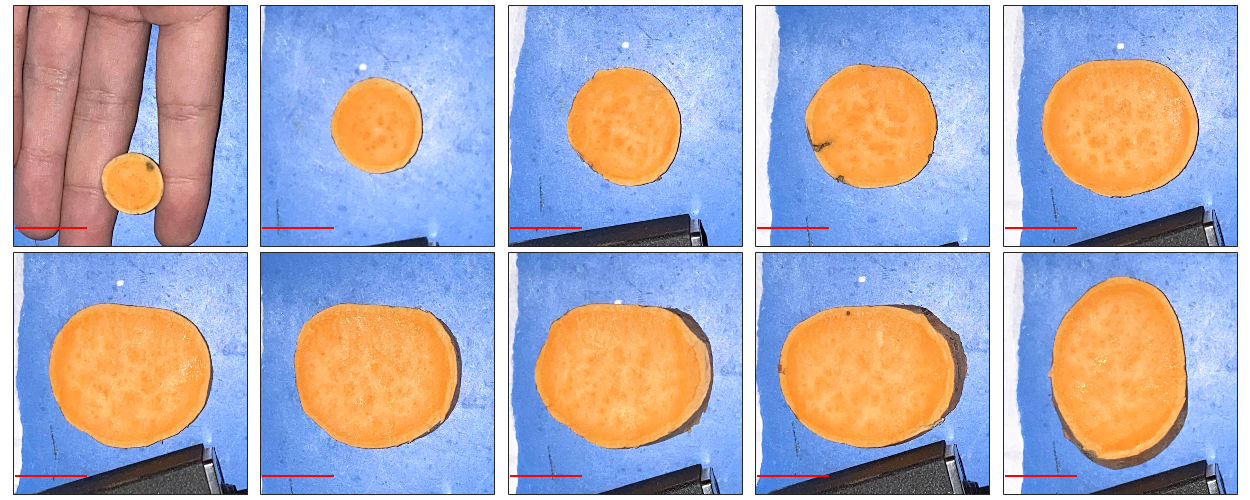


**S1K Fig. RGB imagery of SP1 in batch 2.** Red scale bar indicates 25 mm length. Each panel represents approximately 15±2.3 mm of thickness.


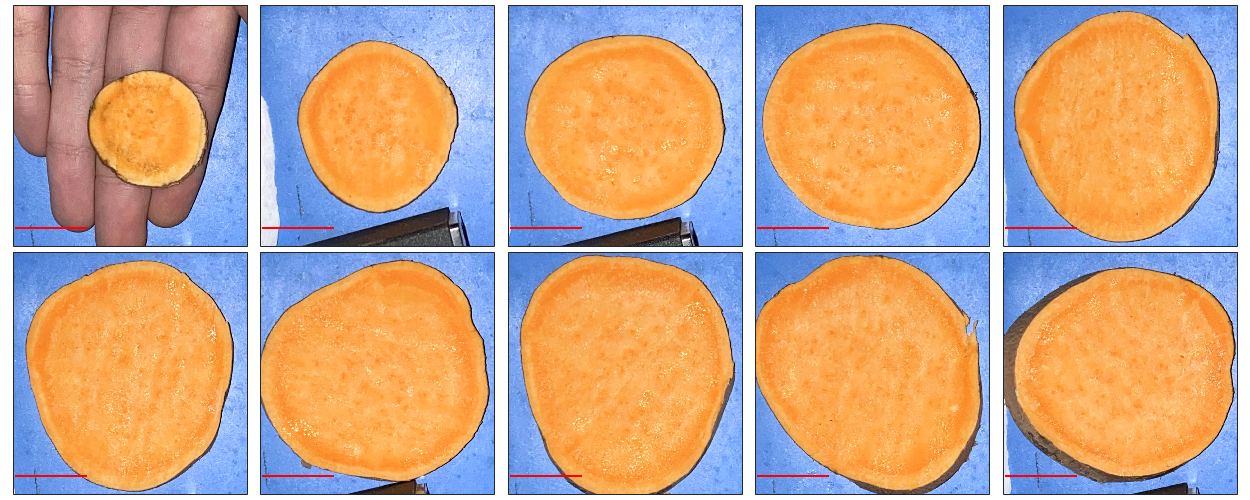


**S1L Fig. RGB imagery of SP2 in batch 2.** Red scale bar indicates 25 mm length. Each panel represents approximately 15±2.3 mm of thickness.

**
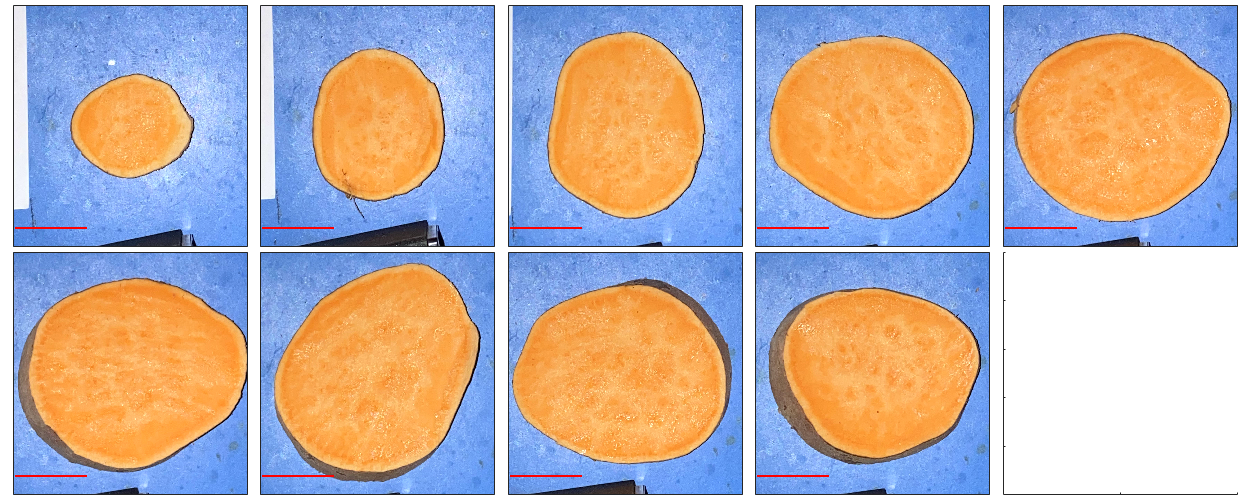
**

**S1M Fig. RGB imagery of SP3 in batch 2.** Red scale bar indicates 25 mm length. Each panel represents approximately 15±2.3 mm of thickness.

**
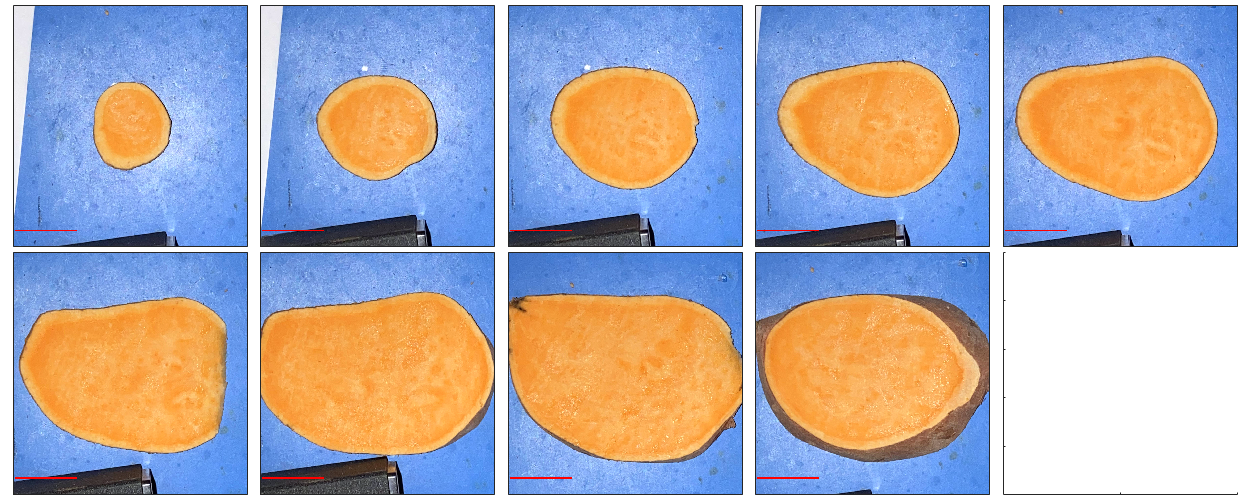
**

**S1N Fig. RGB imagery of SP4 in batch 2.** Red scale bar indicates 25 mm length. Each panel represents approximately 15±2.3 mm of thickness.

**
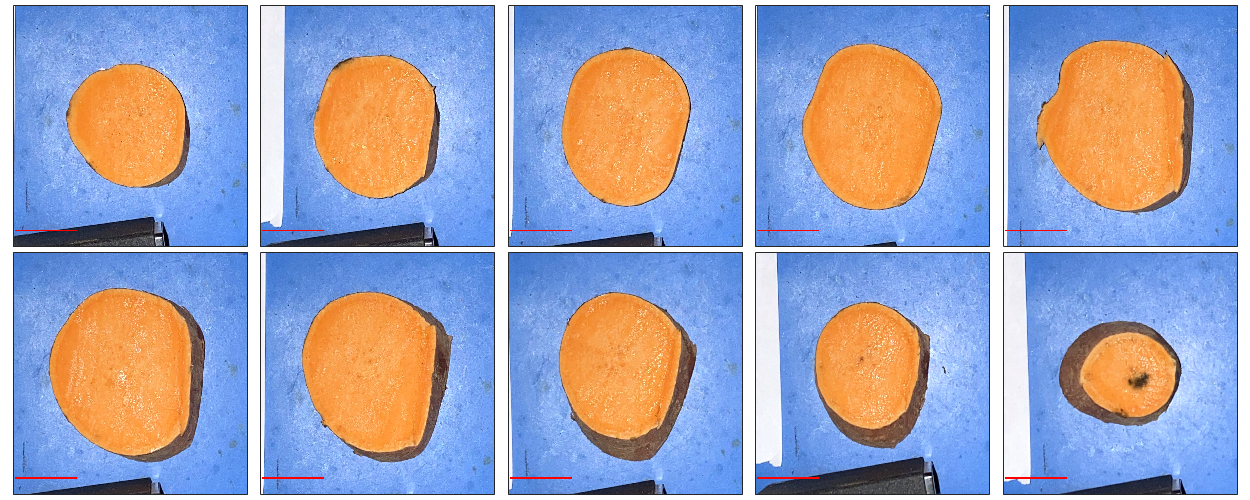
**

**S1O Fig. RGB imagery of SP5 in batch 2.** Red scale bar indicates 25 mm length. Each panel represents approximately 15±2.3 mm of thickness.

**
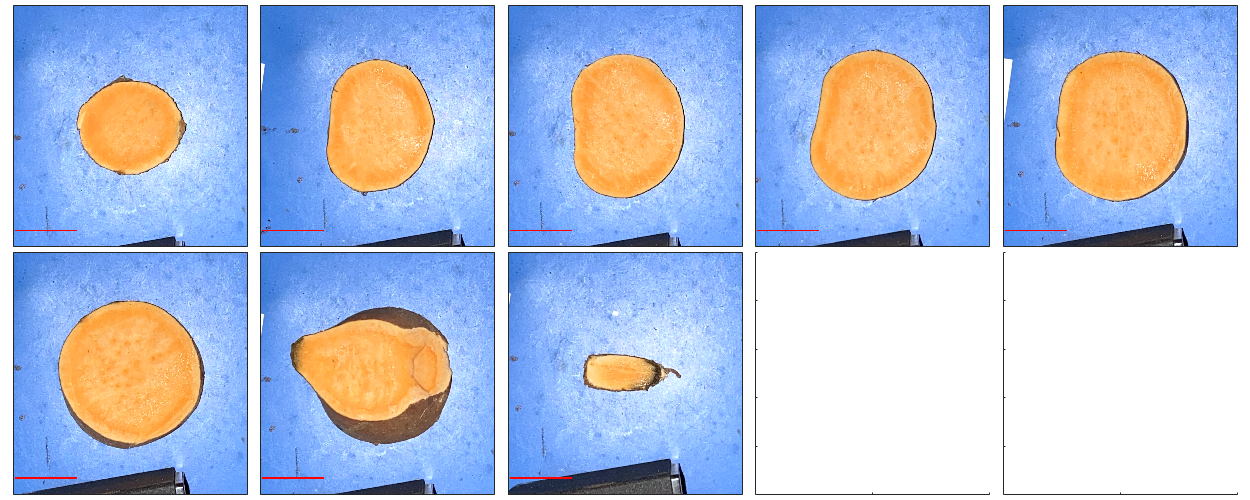
**

**S1P Fig. RGB imagery of SP6 in batch 2.** Red scale bar indicates 25 mm length. Each panel represents approximately 15±2.3 mm of thickness.

**
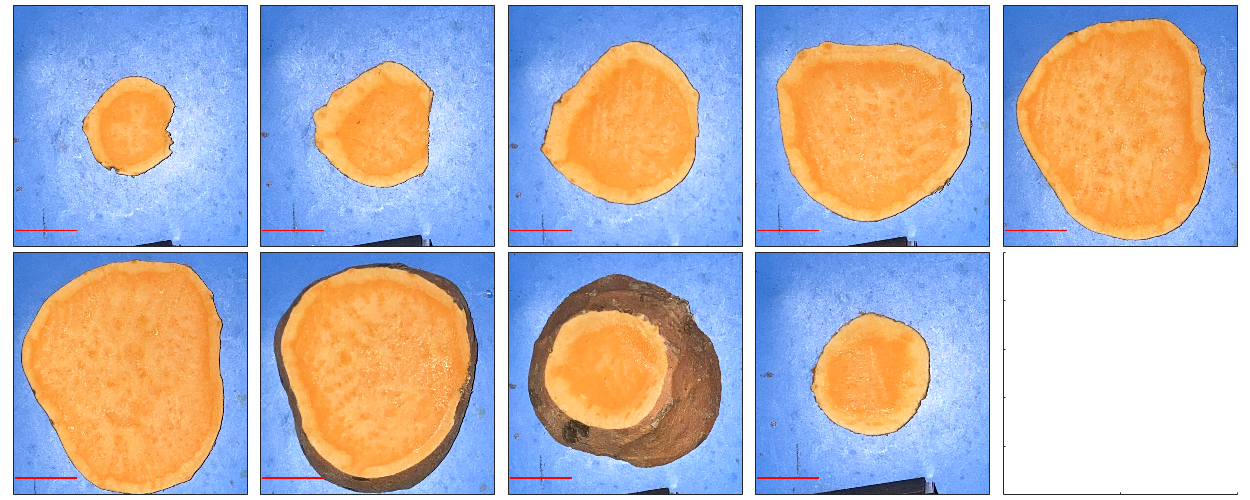
**

**S1Q Fig. RGB imagery of SP7 in batch 2.** Red scale bar indicates 25 mm length. Each panel represents approximately 15±2.3 mm of thickness.

**
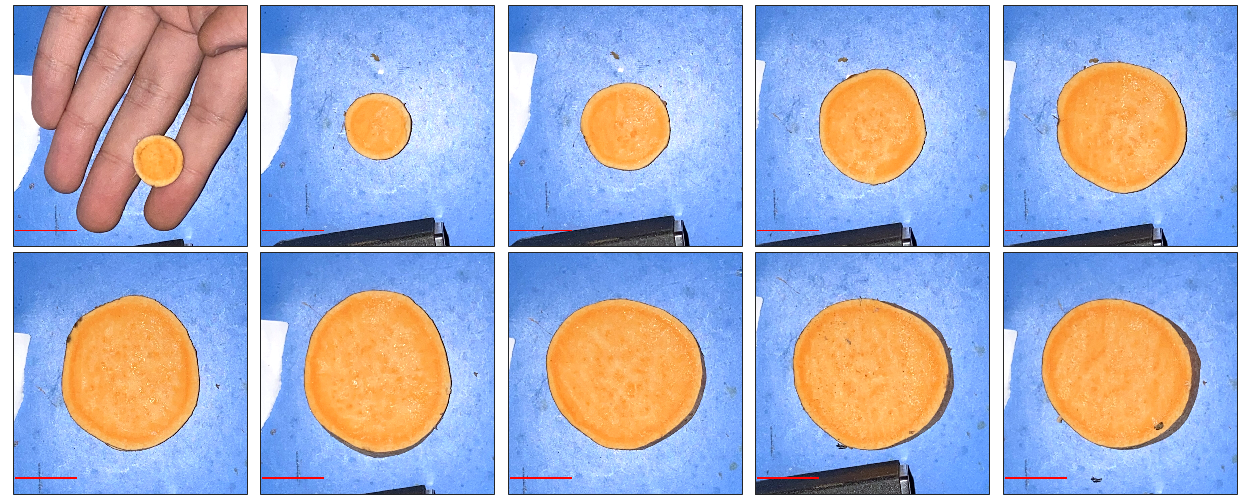
**

**S1R Fig. RGB imagery of SP8 in batch 2.** Red scale bar indicates 25 mm length. Each panel represents approximately 15±2.3 mm of thickness.

**
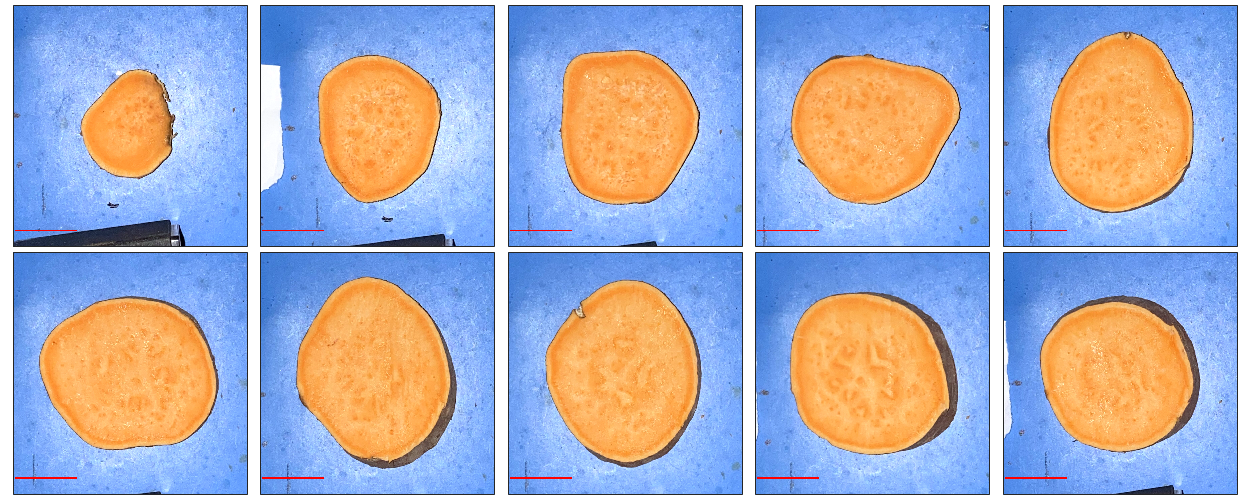
**

**S1S Fig. RGB imagery of SP9 in batch 2.** Red scale bar indicates 25 mm length.

**
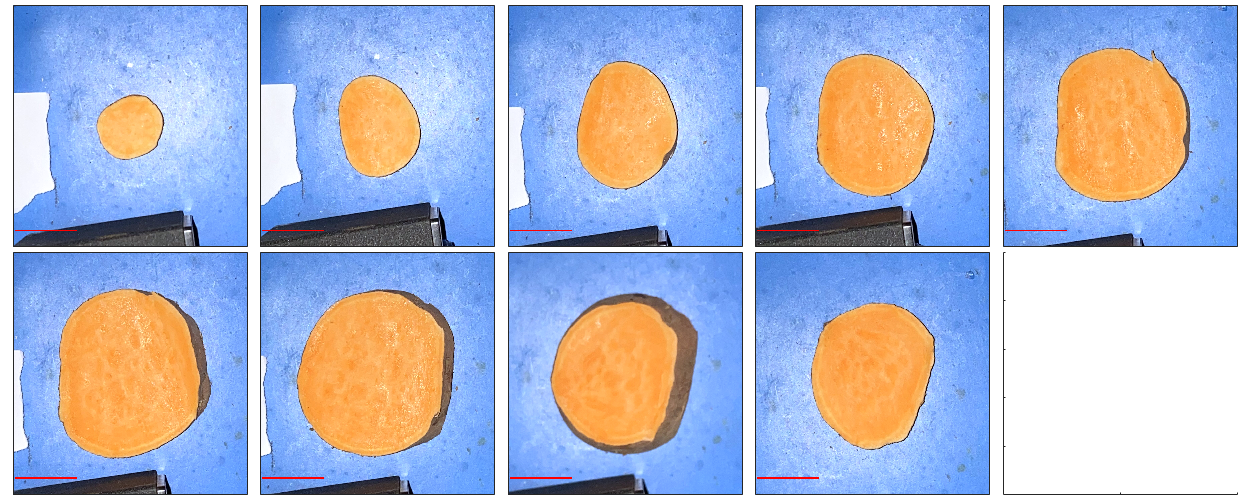
**

**S1T Fig. RGB imagery of SP10 in batch 2.** Red scale bar indicates 25 mm length.

**
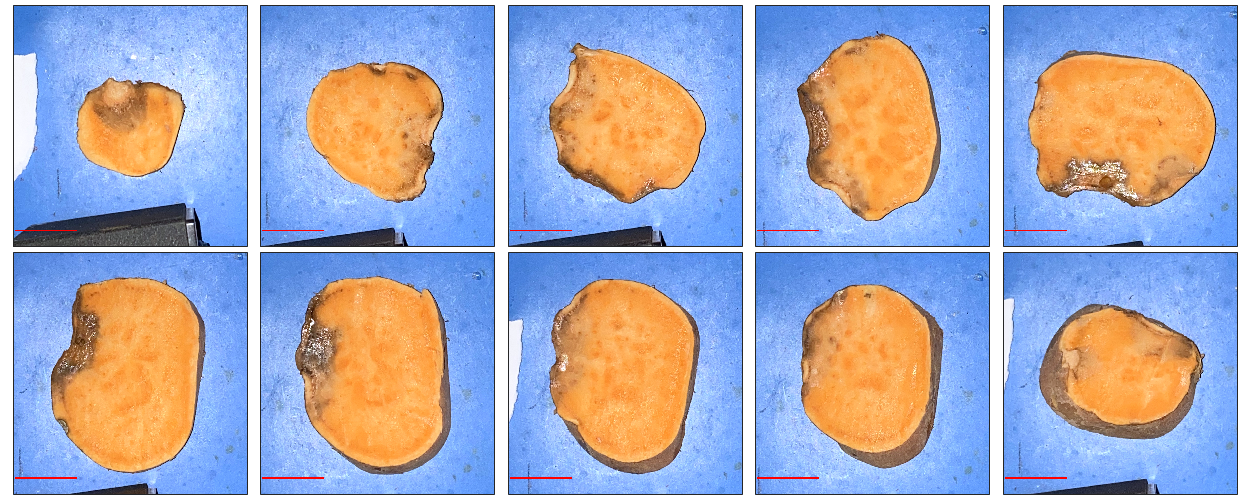
**

**S1U Fig. RGB imagery of SP11 in batch 2.** Red scale bar indicates 25 mm length.

**
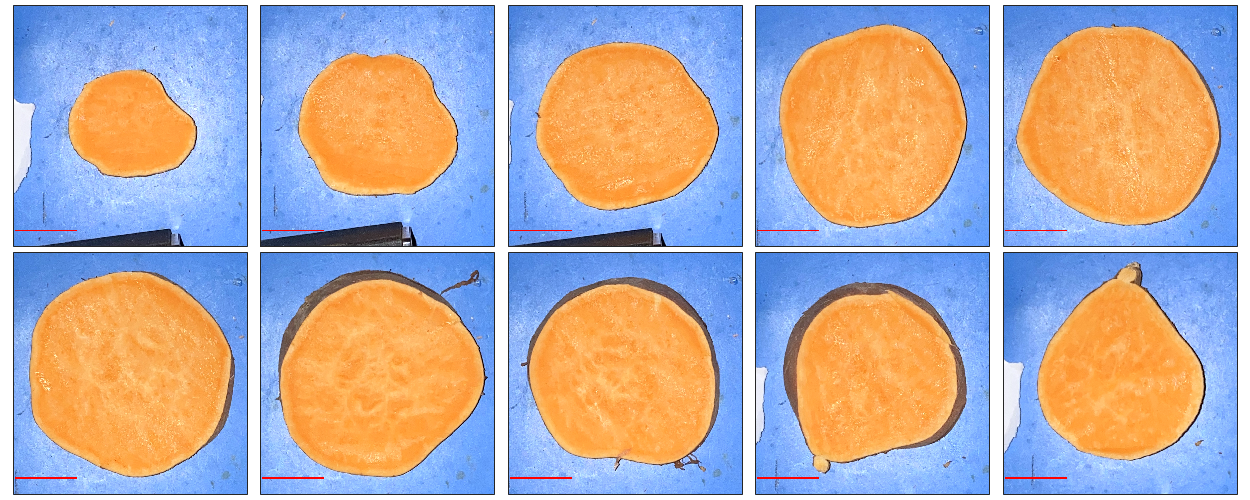
**

**S1V Fig. RGB imagery of SP12 in batch 2.** Red scale bar indicates 25 mm length.
